# Can productivity and profitability be enhanced in intensively managed cereal systems while reducing the environmental footprint of production? Assessing sustainable intensification options in the breadbasket of India

**DOI:** 10.1016/j.agee.2017.10.006

**Published:** 2018-01-15

**Authors:** Virender Kumar, Hanuman S. Jat, Parbodh C. Sharma, Balwinder-Singh, Mahesh K. Gathala, Ram K. Malik, Baldev R. Kamboj, Arvind K. Yadav, Jagdish K. Ladha, Anitha Raman, D.K. Sharma, Andrew McDonald

**Affiliations:** a Crop and Environmental Sciences Division, International Rice Research Institute (IRRI), Los Baños, Philippines; b ICAR-Central Soil Salinity Research Institute, Karnal, Haryana, India; c International Maize and Wheat Improvement Center (CIMMYT)-India Office, New Delhi, India; d International Maize and Wheat Improvement Center (CIMMYT)-Bangladesh Office, Dhaka, Bangladesh; e CCS Haryana Agricultural University, Hisar, Haryana, India; f International Rice Research Institute (IRRI)-India Office, Hyderabad, India; g International Maize and Wheat Improvement Center (CIMMYT)-Nepal Office, Kathmandu, Nepal

**Keywords:** Sustainable intensification, Zero-tillage, Global warming potential, Terminal heat stress, Direct-seeded rice, Sustainability

## Abstract

In the most productive area of the Indo-Gangetic Plains in Northwest India where high yields of rice and wheat are commonplace, a medium-term cropping system trial was conducted in Haryana State. The goal of the study was to identify integrated management options for further improving productivity and profitability while rationalizing resource use and reducing environmental externalities (i.e., “sustainable intensification”, SI) by drawing on the principles of diversification, precision management, and conservation agriculture. Four scenarios were evaluated: Scenario 1 – “business-as-usual” [conventional puddled transplanted rice (PTR) followed by (*fb*) conventional-till wheat]; Scenario 2 – reduced tillage with opportunistic diversification and precision resource management [PTR *fb* zero-till (ZT) wheat *fb* ZT mungbean]; Scenario 3 – ZT for all crops with opportunistic diversification and precision resource management [ZT direct-seeded rice (ZT-DSR) *fb* ZT wheat *fb* ZT mungbean]; and Scenario 4 – ZT for all crops with strategic diversification and precision resource management [ZT maize *fb* ZT wheat *fb* ZT mungbean]. Results of this five-year study strongly suggest that, compared with business-as-usual practices, SI strategies that incorporate multi-objective yield, economic, and environmental criteria can be more productive when used in these production environments. For Scenarios 2, 3, and 4, system-level increases in productivity (10–17%) and profitability (24–50%) were observed while using less irrigation water (15–71% reduction) and energy (17–47% reduction), leading to 15–30% lower global warming potential (GWP), with the ranges reflecting the implications of specific innovations. Scenario 3, where early wheat sowing was combined with ZT along with no puddling during the rice phase, resulted in a 13% gain in wheat yield compared with Scenario 2. A similar gain in wheat yield was observed in Scenario 4 vis-à-vis Scenario 2. Compared to Scenario 1, wheat yields in Scenarios 3 and 4 were 15–17% higher, whereas, in Scenario 2, yield was either similar in normal years or higher in warmer years. During the rainy (*kharif*) season, ZT-DSR provided yields similar to or higher than those of PTR in the first three years and lower (11–30%) in Years 4 and 5, a result that provides a note of caution for interpreting technology performance through short-term trials or simply averaging results over several years. The resource use and economic and environmental advantages of DSR were more stable through time, including reductions in irrigation water (22–40%), production cost (11–17%), energy inputs (13–34%), and total GWP (14–32%). The integration of “best practices” in PTR in Scenario 2 resulted in reductions of 24% in irrigation water and 21% in GWP, with a positive impact on yield (0.9 t/ha) and profitability compared to conventional PTR, demonstrating the power of simple management changes to generate improved SI outcomes. When ZT maize was used as a diversification option instead of rice in Scenario 4, reductions in resource use jumped to 82–89% for irrigation water and 49–66% for energy inputs, with 13–40% lower GWP, similar or higher rice equivalent yield, and higher profitability (27–73%) in comparison to the rice-based scenarios. Despite these advantages, maize value chains are not robust in this part of India and public procurement is absent. Results do demonstrate that transformative opportunities exist to break the cycle of stagnating yields and inefficient resource use in the most productive cereal-based cropping systems of South Asia. However, these SI entry points need to be placed in the context of the major drivers of change in the region, including market conditions, risks, and declining labor availability, and matching with the needs and interests of different types of farmers.

## Highlights

• Higher cereal productivity can be achieved with lower environmental footprint through conservation agriculture.
• Wheat productivity and profitability can be increased by zero-tillage and early sowing.
• Kharif maize appears to be a suitable and profitable alternative to rice in northwest India.
• Productivity and resource efficiency of transplanted rice can be improved by BMPs.
• Directly sown rice has potential to save water, energy and global warming potential compared to transplanted rice.

## 1 Introduction

The rice–wheat cropping system occupies 13.5 Mha in the Indo-Gangetic Plains (IGP) of South Asia, 10.3 Mha of which are in the Indian IGP. This cropping system provides staple food for more than a billion people and is crucial in ensuring food security and livelihood in the region (Chauhan et al., 2012). Sustaining and increasing the production of cereal systems in the Indian states of Punjab, Haryana, and western Uttar Pradesh in the northwest (NW) IGP, together known as the “breadbasket” of the country, are essential to meet the food requirement of India’s burgeoning population, which is likely to increase from 1.3 billion in 2015 to 1.6 billion by 2050.

This cropping system in the NW IGP achieved high productivity during the early Green Revolution period. However, in recent years, the yields of rice and wheat have either stagnated or started to decline along with a decline in total factor productivity (grain output divided by quantity of total input) and profitability, and high inefficiencies in input use (Ladha et al., 2003, Ladha et al., 2009). On the other hand, it is projected that, to feed a population of 1.6 billion, India would have to double its cereal production to meet the food demand by 2050 (Swaminathan and Bhavani, 2013). The challenge is to meet this target using fewer resources (land, water, labor, and chemicals) and with a lower environmental footprint while buffering the risks of climate variability (e.g., erratic rainfall, terminal heat) to ensure long-term sustainability.

The current agricultural production practices in the rice–wheat systems in the NW IGP are neither sustainable nor environmentally sound under the ongoing economic and environmental drivers of agricultural change occurring in the region (Bhatt et al., 2016, Ladha et al., 2009). Current practices require large amounts of resources (labor, water, energy, and biocide) with low input-use efficiencies. At the same time, these resources are becoming scarce and expensive, making conventional practices less profitable and sustainable. For example, rice is predominantly established by the conventional method of puddling and transplanting (PTR) in which rice seedlings are transplanted from the nursery into puddled (wet-tilled) soil in the main field, which is kept flooded for the majority of the growing period (Kumar and Ladha, 2011). This method provides multiple benefits, including good weed control and crop establishment, reduced percolation losses of water, and increased nutrient availability (Johnson and Mortimer, 2005, Sharma et al., 2003), and it is the preferred rice establishment method if labor and water resources are abundant and cheaply available. However, PTR is highly labor-, water-, and energy-intensive as large amounts of labor (for seedling uprooting and transplanting), irrigation water (for puddling and continuous flooding), and energy (for intensive tillage and in irrigation) are needed. Moreover, this production system emits a significant amount of methane (CH_4_) – an important greenhouse gas (GHG) responsible for global warming (Reiner and Milkha, 2000). Furthermore, puddling operations done during rice land preparation can have a negative impact on the yields of succeeding non-rice upland crops (e.g., wheat yield reduction by 8–10%) in the rotation through their negative impact on soil physical properties (Kumar and Ladha, 2011, Kumar et al., 2008). Similarly, conventional practices for wheat consist of intensive land preparation involving multiple passes of discs/tine harrows and planking to create a friable seedbed. This intensive tillage operation leads to a long turnaround period; most often, it resuled. This intensive tillage operation leads to a long turnaround period; most often, it results in a delay in wheat planting, with a yield loss of 27 kg ha^−1^ day^−1^ with every day delay in wheat planting beyond November 15 (Tripathi et al., 2005). Prior to the establishment of rice and wheat, all crop residues (rice and wheat) from the previous crop are either removed for fodder or are burned. However, residue burning results in environmental pollution, nutrient loss (100% C, 90% N, 60% S, and 25% each of P and K) (Dobermann and Fairhurst, 2002), and GHG emissions, with estimates of 110, 2306, 2, and 84 Gg of CH_4_, carbon monoxide (CO), nitrous oxide (N_2_O), and nitrogen oxides (NOx), respectively, in India (Gupta et al., 2004).

To address these problems confronting the rice–wheat system, several improved management practices have been developed under the frameworks of conservation agriculture (CA) or integrated crop and resource management (ICRM) practices (Gathala et al., 2011a, Gathala et al., 2011b, Gathala et al., 2013, Gupta and Seth, 2007, Ladha et al., 2009, Ladha et al., 2016, Laik et al., 2014). These technologies have been developed with the aim of improving the productivity, profitability, and sustainability of rice–wheat systems while reversing resource degradation, improving environmental quality, addressing labor bottlenecks, improving input-use efficiency, and increasing resilience to climate variability. The technologies include reduced or zero-tillage (ZT), laser land leveling, dry direct seeding of rice (DSR), crop residue retention as mulch, site-specific nutrient management, precise irrigation scheduling, and crop diversification (Balwinder-Singh et al., 2011a, Balwinder-Singh et al., 2011b, Gathala et al., 2011a, Gathala et al., 2011b, Kumar et al., 2013, Ladha et al., 2009, Ladha et al., 2016, Sudhir-Yadav et al., 2011a, Sudhir-Yadav et al., 2011b).

ZT in wheat has been widely adopted in NW India, with an area of 0.26 Mha in Haryana State alone (CSISA, 2010), and it is now gaining momentum in the eastern IGP (CSISA, 2015, Keil et al., 2015) mainly because of its clear and positive impacts on productivity, profitability, resource-use efficiency, and resilience to heat stress (Erenstein and Laxmi, 2008, Keil et al., 2015). There is increased interest among government agencies to promote DSR and to diversify rice with maize in an attempt to arrest the declining groundwater table as well as the problem of labor scarcity. DSR combined with ZT was found to reduce labor and irrigation water requirements and to provide more net profit than PTR without any yield penalty (Gathala et al., 2013, Kumar and Ladha, 2011, Laik et al., 2014, Sudhir-Yadav et al., 2011a, Sudhir-Yadav et al., 2011b). Similarly, maize in the monsoon season appears to be a suitable and profitable alternative to rice in the NW IGP as it can overcome problems of rising scarcity of resources (Gathala et al., 2013). Also, the availability of the “Happy Seeder”– a ZT machine that can plant rice and wheat in high-residue (anchored and loose) conditions – has made it possible to retain the residues on the soil surface, thereby providing an alternative to residue burning (Gathala et al., 2011c, Sidhu et al., 2007, Sidhu et al., 2008, Sidhu et al., 2015). To harness the full benefits of CA, ZT in combination with residue retention on the soil surface as mulch has to be integrated with precision management and a more diversified crop rotation. Despite multiple examples of the benefits associated with CA-based practices in South Asia, some recent studies have questioned the role of CA in climate change mitigation as well as the challenges in achieving economic and ecosystem benefits in smallholder farming (Brouder and Gomez-Macpherson, 2014, Palm et al., 2014, Powlson et al., 2014, Pittelkow et al., 2014).

A holistic systems approach and more medium- to long-term studies are needed to evaluate the benefits and trade-offs associated with the adoption of these CA-based best management practices (BMPs). Many of these technologies, either as stand-alone or in combination in a single crop season and in cropping systems, have been evaluated in the region. The short-term impacts of these technologies on productivity and profitability have also been reported by many studies (Bhushan et al., 2007, Gathala et al., 2013, Jat et al., 2009, Laik et al., 2014, Ladha et al., 2016, Saharawat et al., 2010), although only a few studies reported their medium- to long-term performance on a systems basis (Gathala et al., 2011a, Gathala et al., 2011b, Jat et al., 2014, Singh et al., 2011, Singh et al., 2016). Most of these studies have reported yield and economics and some have reported irrigation water use. However, only a few studies have examined CA-based BMPs holistically from a multi-dimensional perspective spanning yield, economic, and environmental impacts along with analyzing the potential trade-offs. Furthermore, even fewer studies have explored the stability of the system with time to document responses to growing season weather variability as well as the cumulative impact of management changes on resource quality with time.

To address these knowledge gaps in the most intensified rice–wheat cropping systems in South Asia, a production-scale research platform was established at the Central Soil Salinity Research Institute (CSSRI), Karnal, Haryana, India, in 2009. The overarching objective of this research platform is to assess the short- to long-term performance of different cereal-based cropping systems within key scenarios of agricultural change using a wide range of indicators (e.g., yield; resource-use efficiency; crop, soil, and environmental health; economics; and energy). The overall goal of the study was to identify a new generation of resource-efficient and high-yielding cereal systems that draw on the principles of CA and precision agriculture. Initial results for the first two years of the experiments demonstrated increases in system productivity, water productivity, and profitability from CA and BMPs relative to conventional practices (Gathala et al., 2013). In this paper, the medium-term (5-year) performance of the system is presented with respect to yield, water use, water productivity, economics, energy requirements, and GWP.

## 2 Materials and methods

### 2.1 Experimental site details

**Fig. 1:**
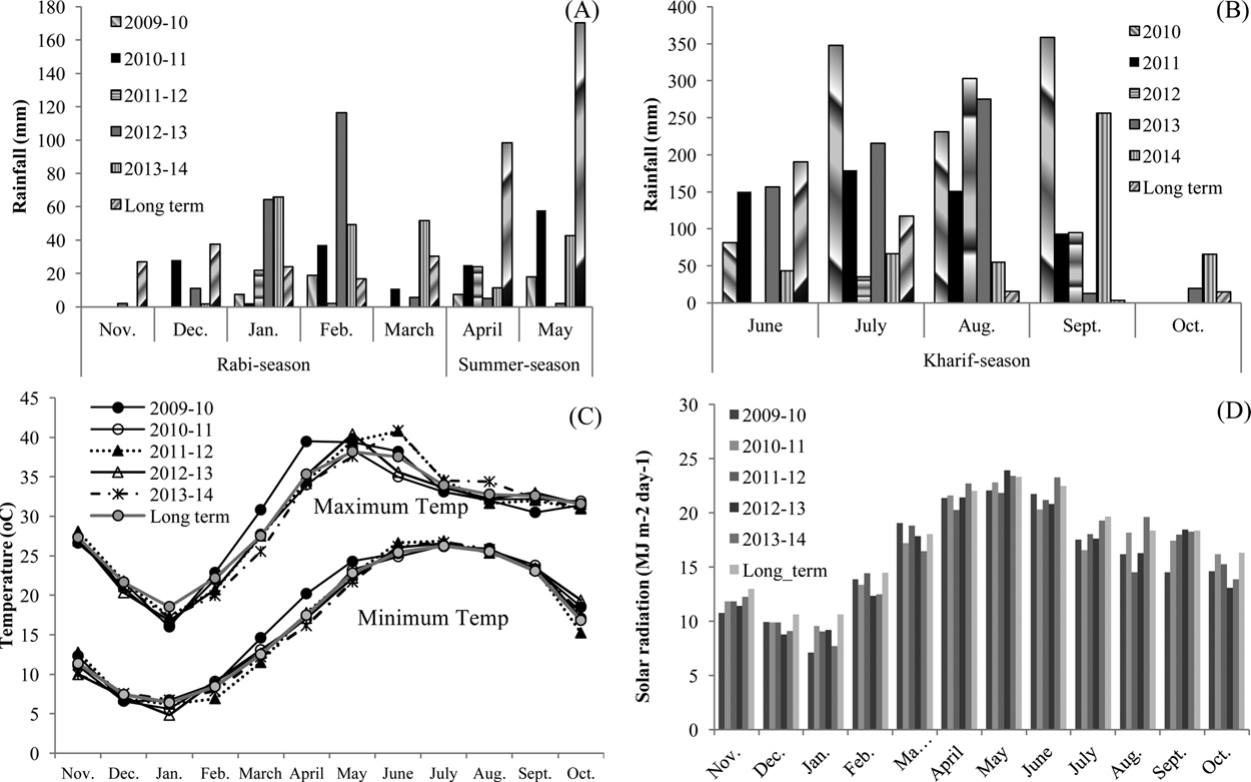
Monthly rainfall for rabi, summer (A), and kharif season (B), monthly average daily maximum and minimum temperature (C), and monthly mean daily solar radiation (D) during study years 2009–10 to 2013–14 along with 30-year long-term average (1982–2012).

Under the umbrella of the Cereal Systems Initiative for South Asia (CSISA) project (www.csisa.cimmyt.org), a long-term production-scale field study was established in 2009 at the CSISA experimental research platform located in the NW IGP (29°70′N, 76°96′E) at CSSRI, Karnal, Haryana (Gathala et al., 2013). Haryana is representative of the irrigated, highly productive, and high-input rice–wheat cropping systems of the NW IGP. The soil at the experimental site is reclaimed alkali with loam texture as per USDA classification. Based on the analysis done in 2009 at the beginning of the experiment, the 0- to 15-cm soil layer had clay, silt, and sand contents of 19.9%, 46.1%, and 34.0%, respectively; pH of 8.0; electrical conductivity of 0.37 dS m^−1^; oxidizable soil organic carbon 0.45%; total N 0.06%; Olsen P 5.74 mg kg^−1^; and 1 M NH_4_OAc extractable K of 130 mg kg^−1^ (Gathala et al., 2013). The climate in Haryana is semi-arid, with an average annual rainfall of 700 mm, 75–80% of which is received between June and September. The daily minimum and maximum temperatures range from 0 to 4 °C in January and from 41 to 44 °C in June, respectively. The relative humidity varies from 50% to 90% throughout the year. The seasonal weather data during the study period (2009–2014), which include rainfall, minimum and maximum temperature, and solar radiation, are shown in Fig. 1.

### 2.2 Experimental design, treatments, and crop management

Four cereal cropping system scenarios (treatments) were established in 2009–10, varying in crop sequence, tillage and crop establishment methods, residue management, and other crop management practices (Gathala et al., 2013). These scenarios were designed to address different drivers of agricultural changes occurring in NW India (see Table 1 for details) and were replicated thrice in production-scale plots of 100 m × 20 m (2000 m^2^) in a randomized complete block design. The full experimental details can be obtained from an earlier published paper (Gathala et al., 2013).

**Table 1:** Details of scenario treatments with drivers of change, crop rotation, tillage, crop establishment methods, residue management, and level of conservation agriculture practices adopted.

Parameter	Scenario 1	Scenario 2	Scenario 3	Scenario 4
Scenario description	Business-as-usual	Reduced tillage, opportunistic diversification, and precision resource management	Zero tillage for all crops, opportunistic diversification, and precision resource management	Zero tillage for all crops, strategic diversification, and precision resource management
Drivers of change	None	Rising production cost and input-use inefficiencies	As in Scenario 2 + rising scarcity of labor, water, energy, and environmental protection	Same as Scenario 3 + diversification to arrest groundwater decline
Goal and approach	Maintaining current productivity and input-use efficiency using current farmers' practice	Optimizing productivity and profitability and input-use efficiencies by using low-risk and proven opportunities for sustainable crop intensification and best management practices (BMPs)	Optimizing productivity and profitability and input-use efficiencies by crop intensification and BMPs using CA-based practices	Optimizing productivity and profitability and input-use efficiencies by crop intensification, diversification, and BMPs using CA-based practices
Crop rotation	Rice-wheat-fallow	Rice-wheat-mungbean	Rice-wheat-mungbean	Maize-wheat-mungbean
Cropping intensity	200%	300%	300%	300%
Tillage	Conventional tillage: *Rice-puddling; wheat- conventional*	Mixed tillage: *Rice-puddling; wheat and mungbean-ZT*	Zero tillage: *Rice, wheat, and mungbean: ZT*	Zero tillage: Maize*, wheat, and mungbean: ZT*
Crop establishment methods	Rice: transplanting; wheat: broadcast	Rice: transplanting; wheat: drill-seeded; mungbean: drill-seeded except in years 2 and 3	Rice and wheat: direct drill-seeded; mungbean: drill-seeded except in years 2 and 3	Maize and wheat: direct drill-seeded; mungbean: drill-seeded except in years 2 and 3
Crop residue recycling in five years (Mg ha^−1^)	Residue removed; only small stubbles incorporated into soil	59 Mg ha^−1^ either retained on soil surface or incorporated	55 Mg ha^−1^ retained on soil surface as mulch	70 Mg ha^−1^ retained on soil surface as mulch
Level of CA	None	Partial CA	Full CA	Full CA

#### 2.2.1 Scenario 1 (Business-as-usual)

This scenario was designed to represent the prevailing crop rotation and management practices employed by farmers in the region; thus, it is known as the business-as-usual scenario. Rice–wheat–fallow is the most dominant cropping system in the region (Table 1); hence, both rice and wheat were established using conventional practices for this experiment: rice by transplanting 30-35-day-old seedlings of the most dominating long-duration variety [Pusa-44; 150 days’ duration (seed to seed)] manually in a random pattern with 1–2 seedlings per hill into puddled soil, and wheat by manual broadcasting in tilled soil around mid-November. Residues of both rice and wheat crops were completely removed. For water management in rice, continuous flooding was applied at a depth of 5 cm for the first month and water was applied at the appearance of a hair-line crack for the remaining period (Gathala et al., 2013). In wheat, irrigation was applied at the following growth stages: crown root initiation, tillering, jointing, flowering, and milk and grain filling.

#### 2.2.2 Scenario 2 (Reduced tillage, opportunistic diversification, and precision resource management)

This scenario evaluates the most ostensibly low-risk and proven opportunities for the sustainable intensification of rice–wheat systems with mungbean added as a third crop in the rotation following wheat. Rice was established by transplanting into puddled soil, but both wheat and mungbean were cultivated with ZT. The 25-day-old rice seedlings (younger than those used in Scenario 1) of a medium-duration hybrid [Arize 6444; 130–135 days’ duration (seed to seed)] were manually transplanted into puddled soil with 1–2 seedlings per hill in lines at 20 × 15 cm spacing. Wheat and mungbean were drill-sown under ZT conditions with a row spacing of 20 cm using the Happy Seeder with full retention of the residue from the previous crop at the soil surface. Wheat was sown at the same time as in Scenario 1. An efficient water management practice known as alternate wetting and drying (AWD) was applied in rice, which consists of (1) continuous flooding at a depth of 5 cm for the first 15–20 days after transplanting, (2) irrigation when the soil dried to 40 to 50 kPa matric potential at 15-cm soil depth until 1 week before flowering, and (3) irrigation at 15–20 kPa for the remainder of the cropping season. Water management in wheat was similar to that used in Scenario 1 where irrigation was applied at critical growth stages.

#### 2.2.3 Scenario 3 (ZT for all crops, opportunistic diversification, and precision resource management)

This scenario is the same as Scenario 2 (rice–wheat–mungbean) but with all three crops drill-sown under ZT conditions, thereby attempting to greatly reduce system-level energy, water, and labor requirements by transitioning away from PTR. For DSR, a shorter-duration rice hybrid [Arize-6129; 115–120 days’ duration (seed to seed)] was used and wheat was sown earlier (by the end of October) than in Scenarios 1 and 2 as the rice fields were vacated early by the short-duration rice cultivar. For water management in rice, the soil was kept moist but not flooded for the first 20 days to ensure good crop establishment and then irrigation was applied at 15–20 kPa soil tension at 15-cm soil depth, which is considered safe for non-puddled DSR systems in this region (Sudhir-Yadav et al., 2011a, Sudhir-Yadav et al., 2011b, Gathala et al., 2013). Water management in wheat was similar to that applied in Scenarios 1 and 2.

#### 2.2.4 Scenario 4 (ZT for all crops, strategic diversification, and precision resource management)

This scenario was designed to explore maize as an option for diversifying away from rice (maize–wheat–mungbean system) and this requires less energy, labor, and irrigation water, even when contrasted with ZT rice in Scenario 2 (Table 1). Wheat was sown early (by the end of October) as in Scenario 3. All three crops (maize, wheat, and mungbean) in this scenario were drill-sown under ZT conditions. Irrigation in maize was applied if needed to avoid any moisture stress at critical growth stages, including early vegetative, tasseling, and silking, and during the remaining growing season. Irrigation was applied based on visual observations (any sign of curling of leaves); in wheat, it followed the application strategies used in Scenarios 1–3.

Other crop management practices, including land preparation, cultivars used, seed rate, sowing date, seed treatment, and nutrient and pest management in rice, wheat, and maize under different scenarios, are given in Table 1 of the Supplementary data.

### 2.3 Residue management and estimation of crop residues recycled

All previous crop residues were removed in Scenario 1 except for the small stubbles that were left after crop harvest at ground level, which were incorporated with tillage. In Scenario 2, all rice residues except those in Years 1 and 2, which were only partially anchored, were retained at wheat sowing while the anchored wheat stubbles (∼15 cm in height) were retained at mungbean sowing. All mungbean residues were incorporated during the puddling operation in rice. In Scenario 3, all rice and mungbean residues and the anchored wheat stubbles (∼15 cm in height) were retained on the soil surface. Similarly, in Scenario 4, either full (in Year 1) or partial (65% in Years 2–5) maize residues, full mungbean residues, and anchored wheat stubbles (∼15 cm in height) were retained at the soil surface.

To estimate the amount of crop residue recycled in each scenario, five rows with a length of 1 m were sampled from four locations in each plot after the harvest of each crop. The residues were cut from the soil surface, oven-dried, and expressed on a dry weight basis per hectare. Over the five-year period (2009–14), 59, 55, and 70 t ha^−1^ of crop residues were retained or incorporated for Scenarios 2, 3, and 4, respectively (Table 1).

### 2.4 Crop harvest and yield estimation

Rice was harvested and threshed either manually or using a combine harvester at a height of 30 cm above ground level (Scenarios 2 and 3) or at ground level (Scenario 1). Wheat was harvested either by a combine or by a reaper and binder at about 15 cm above ground level in all scenarios except Scenario 1, in which the wheat was harvested at ground level. The wheat was then threshed using a plot thresher. In the case of maize, the cobs were picked manually and threshed using a maize sheller. At maturity, the grain and straw yields of both wheat and rice were determined on a total area of 100 m^2^ by sampling from four locations of 25 m^2^ each. Grain and straw yields of maize were estimated by harvesting a total area of 120 m^2^ from each plot by sampling from four locations of 30 m^2^ each. Grain moisture content was determined by using a grain moisture meter at the time of yield estimation and grain yield was expressed as t ha^−1^ at 12% (wheat) or 14% (rice and maize) moisture content. For mungbean yield estimation, the entire plot was harvested and weighed. To compare the productivity of different crops and total system productivity of the different scenarios, the yield of non-rice crops (wheat, maize, and mungbean) was converted into rice equivalent yield (REY) (t ha^−1^) and calculated as follows:

$$\text{Rice equivalent yield of non-rice crop=}\frac{\text{Yield of non-rice crop}\;\;\left({\text{kg ha}}^{-1}\right)\text{* price of non-rice crop}\;\;\left(INR\;\;{kg}^{-1}\right)}{\text{Price of rice}\;\;\left(INR\;\;{kg}^{-1}\right)}$$

The prices used were the minimum support prices of rice, wheat, maize, and mungbean crops guaranteed by the government in the respective years.

### 2.5 Water measurement

During the rainy (*kharif*) season, irrigation was applied based on tensiometer readings in Scenarios 2 and 3. To monitor the soil matric potential (SMP), gauge-type soil tensiometers (IRROMETER, Riverside, California) were installed at 15-cm and 30-cm depths in all plots immediately after each crop was planted. During the winter (*rabi*) season, water was applied based on the wheat growth stages. Irrigation water applied to each plot was measured using a Woltman^®^ turbine water meter. For more details on irrigation pipe installation, please refer to Gathala et al. (2013). The total irrigation input to each crop (including pre-sowing irrigations) and to the total system each year was determined. To measure the amount of water applied in each irrigation, the water meter reading (kiloliter, kL) was recorded at the start and end of the irrigation of each plot. For all crops in all scenarios, each irrigation was applied until a 5-cm flooding depth was achieved. The amount of irrigation water applied was calculated as water depth (mm) (Gathala et al., 2013). Water productivity with respect to irrigation (WP_I_) and total water input (irrigation + rain, WP_I+R_) were calculated:

$${\text{WP}}_{1}(\text{kg}\;\text{ g}\text{rain}\;\;{\;\text{m}}^{-3}\;\;\text{o}\text{f}\;\;\text{ir}\text{rig}\text{ati}\text{on}\;\;\text{ water})=\left(\frac{\text{Grain}\;\text{ yield}\;\;(\text{kg}\;\text{ h}{\text{a}}^{-1})}{\text{irrigation}\;\;(\text{mm})}\right)/10$$

$${\text{WP}}_{1+\text{R}}(\text{kg}\;\text{ grain}\;\;{\;\text{m}}^{-3}\;\;\text{ of}\;\text{ irrigation}\;+\text{rain}\text{wa}\text{ter})=\left(\frac{\text{Grain}\;\;\text{ yield}\;\;(\text{kg}\;\;\text{h}{\text{a}}^{-1})}{\text{irrigation+rainfall}\;\;(\text{mm})}\right)/10$$

### 2.6 Economic analysis

All fixed and variable costs were considered in the economic analysis (Table 2 in Supplementary data). The variable costs (excluding land rent) consisted of labor cost; costs of other inputs such as tillage, planting, seed, fertilizer, pesticide, irrigation, harvesting, threshing, etc.; and costs involved in transporting grains to the market. Fixed costs consisted of the depreciation of machinery and interest on working capital. The cost of human labor used for tillage, seeding, irrigation, fertilizer and pesticide application, weeding, and harvesting of crops was based on person-days ha^−1^. The time (h) required to complete each field operation in each treatment was also recorded and expressed as person-days ha^−1^, considering 8 h to be equivalent to 1 person-day (standard working hours as per the labor law of the Indian government). The cost of labor was calculated using the minimum wage rate as per the labor law (Minimum Wage Act, 1948). Similarly, the time (h) required by a tractor-drawn machine/implement to complete a field operation such as tillage, seeding, and harvesting was recorded and expressed as h ha^−1^. For irrigation costs, the charges fixed by the electricity board of the Haryana Government (INR 0.30 per kW h of electricity) were used plus the cost of labor used for irrigation application. Gross returns (GR) were calculated by multiplying the grain yield of each crop by the minimum support price offered by the Government of India (Economic Survey of India, various years), while straw value was calculated using current local market rates. Net returns (NR) were calculated as the difference between GR and total cost (TC) (NR = GR − TC). System net returns (SNR) were calculated by adding the net returns of crops for the crops harvested within an individual calendar year. The benefit:cost ratio (B:C ratio) was calculated by dividing gross income by TC (B:C ratio = GR/TC).

### 2.7 Energy estimation

To estimate the total energy used in each crop and each cropping system under the different scenarios, the energy equivalent (MJ unit^−1^) of each input was used (Table 2). Fuel consumed during each field operation, including land preparation, seeding, harvesting, and threshing, was recorded to calculate the energy consumption. Energy usage for irrigation was estimated by recording the number of hours the electric pump was used. Similarly, the energy requirement for all other inputs was estimated based on actual practices (i.e., activity and hours of use) using the energy equivalent values in Table 2. The specific energy (MJ kg^−1^) required to produce grain was calculated as the ratio of the total energy input (MJ ha^−1^) to the grain yield (kg ha^−1^) produced. For energy budgeting, total energy output was also estimated using grain and straw yield as output. Net energy return was calculated by subtracting total energy input from total energy output.

**Table 2:** Energy equivalent values for various inputs.

Particulars	Unit	Energy equivalent (MJ unit^−1^)	Reference
Human labor	Person-h	1.96	Shahin et al. (2008)
Diesel fuel	Liter	56.31	Shahin et al. (2008)
**Chemical fertilizers**	kg		
Nitrogen		66.14	Shahin et al. (2008)
Phosphorus (P_2_O_5_)		12.44	Shahin et al. (2008)
Potassium (K_2_O)		11.15	Shahin et al. (2008)
Zinc		8.4	Shahin et al. (2008)
**Pesticide**	kg ai ha^−1^		
Herbicide		238	Gündoğmuş (2006)
Insecticide		199	Gündoğmuş (2006)
Fungicide		92	Gündoğmuş (2006)
**Seeds**	kg	15.2	Rahman and Rahman (2013) and Yadav et al. (2013)
Electricity^a^	kWh	11.93	Mobtaker et al. (2010)
**Outputs**	kg		
Grain yield		14.7	Shahin et al. (2008)
Straw yield		12.5	Shahin et al. (2008)

^a^A 1-HP electric motor consumes 0.746 kW, so a 15-HP motor used for pumping water will consume = 15 × 0.746 = 11.2 kW of energy in 1 h.

### 2.8 Global warming potential (GWP) estimation

The total GWP [carbon dioxide (CO_2_) equivalent] of each crop and each scenario was calculated by adding the estimated GWP emission factors (Table 3) associated with the production of all agricultural inputs including fertilizers, pesticides (herbicides, insecticides, and fungicides), electricity, and diesel fuel. In addition to these sources, season-long CH_4_ and N_2_O fluxes were estimated using emission patterns for rice, wheat, and maize under different management practices based on field measurements made at the experimental station in the present study as well as from companion trials at the same experimental station and in nearby farmers’ fields (Padre et al., 2016). The emission factors for N_2_O (% of applied N) for rice, wheat, and maize were 0.51, 0.33, and 1.95, respectively, whereas the CH_4_ emission factors for PTR, DSR, wheat, and maize were 12.8, 5.6, 0, and 0 kg ha^−1^ season^−1^, respectively (Padre et al., 2016).

**Table 3:** Greenhouse gas (GHG) emission factors for different agricultural inputs.

Input	Emission factor (kg CO_2_ eq. per unit of input)	Source
Diesel fuel	2.68 l^−1^	US-EIA (2011): www.eia.gov/oiaf/1605/emission_factors.html (Fuel Emission Factors)
Electricity	0.994 kW h^−1^	
N (kg)	4.95	CFT (2014)
P (kg)	0.73	CFT (2014)
K (kg)	0.545	CFT (2014)
Average pesticide	26.63 kg^−1^ a.i.	Audsley et al. (2009)
Average herbicide	24.20 kg^−1^ a.i.	Grassini and Cassman (2012)
Emission factor for N_2_O emissions (kg N applied) for rice	0.51	Padre et al. (2016)
Emission factor for N_2_O emissions (kg N applied) for wheat	0.33	Padre et al. (2016)
Emission factor for N_2_O emissions (kg N applied) for kharif maize	1.95	Padre et al. (2016)
Methane emission factor for puddled transplanted rice (kg season^−1^)	12.8	Padre et al. (2016)
Methane emission factor for dry-seeded rice (kg season^−1^)	5.6	Padre et al. (2016)

For calculating N_2_O emissions per season, the following equation was used:

$$\text{Season long}N_{2}O\text{ emission}=\frac{\left[N_{2}\text{O emission factor}\left(\text{\% of N applied}\right)\right]/100\text{ x}44}{28}\text{ x total N applied to the crop}$$

### 2.9 Statistical analysis

Year-wise and across-year scenario means were calculated for grain yields, water application, energy budgeting, economics, and GWP. The linear mixed model for the observation *y_ijk_* of the *j*th replicate of the *i*th treatment at time (year) *k* is:

$$y_{ijk}=\mu +\alpha_{i}+\tau_{k}+{\left(\alpha \tau \right)}_{ik}+\varepsilon_{ijk}$$

where $\alpha_{i},\tau_{k},{\left(\alpha \tau \right)}_{ik}$ were the *fixed* effects of treatment *i*, time *k*, and their interaction, respectively, and *ε*_*ijk*_ is the random error associated with the *jth* replicate of the *ith* treatment at year *k*. The ante-dependence covariance structure was fitted to the errors to model the serial correlation across years (Resende et al., 2006, Zimmerman and Núñez-Antón, 2009). This implies that the error terms *ε*_*ijk*_ from the *jth* replicate of the *ith* treatment across years *1…k* are correlated and the correlation is a function of their distance in time – where adjacent observations tend to be more highly correlated than distant observations. The correlation between two non-adjacent elements is the product of the correlations between the elements that lie between the elements of interest. This structure requires t + (t − 1) parameter estimates. With this structure, time periods must be ordered correctly and equal spacing between times is not necessary (Wang and Goonewardene, 2004). The covariance matrix is expressed as:
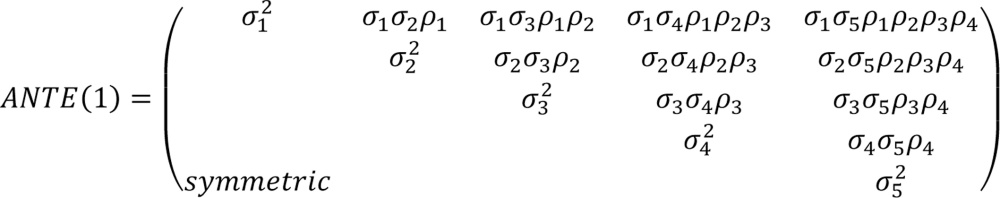
where the subscripts refer to the years. The correlations between the scenario plots across years within a replicate are assumed to be the same for all scenarios.

Relative yields of wheat, rice/maize, and system in Scenarios 3 and 4 in comparison with that in Scenario 2 and also in Scenarios 2, 3, and 4 in comparison with that in Scenario 1 were computed over the five years and trend analysis was done using linear regression. Relative yields were computed as given below:

Relative yields in Scenario 3 or 4 in comparison to Scenario 2 = yield in Scenario 3 or 4/yield in Scenario 2

Relative yields in Scenario 2 or 3 or 4 in comparison to Scenario 1 = yield in Scenario 2 or 3 or 4/yield in Scenario 1

## 3 Results and discussion

### 3.1 Weather data

Wheat season: The weather during the 2012–13 and 2013–14 wheat season was wetter, mainly due to unusually higher rainfall in January and February in 2012–13 (181 mm versus 41 mm) and from January to March in 2013–14 (167 mm versus 71 mm) compared with the long-term average (Fig. 1A). These two years were wetter than the other three years during the study period. The first three wheat seasons were relatively drier than the long-term average, especially 2009–10 and 2011–12 with seasonal rainfall of 27 and 24 mm, respectively, compared with the long-term average seasonal rainfall of 136 mm. Mean monthly maximum and minimum temperatures were similar to the long-term values, apart from higher minimum and maximum values in March 2009–10, higher minimum temperature in February and March 2010–11, and lower minimum temperature in February and March 2011–12 and lower maximum temperature in February and March 2013–14 (Fig. 1C). Monthly mean daily pan evaporation (Epan) was similar during most of the growing season in all the years (data not shown). Monthly mean daily solar radiation was in general lower in all years than the long-term average except in February and March 2011–12 and in March 2009–10 (Fig. 1D). Within the study years, solar radiation from January to March was lowest in the 5th year.

Rice season: During the rice/maize season (June–October), the amount of rainfall was much higher in 2010 (1018 mm) and was lowest in 2012 (432 mm) than in other years during the study period (Fig. 1B). The monthly average daily temperature was similar in all the years except for the high temperatures recorded in June, July, and August in 2014 compared with the long-term average and other study years (Fig. 1C). There was large variability in solar radiation value during the study period (Fig. 1D). Solar radiation was lower than the long-term average from July to October 2009–10, in July 2010–11 and 2012–13, in August 2011–12 and 2012–13, and in October 2012–13 and 2013–14.

### 3.2 Crop and system yields

#### 3.2.1 Wheat

Based on the five-year average, Scenarios 3 and 4 with full CA-based management practices yielded 0.90 to 0.95 t ha^−1^ (13–19%) higher than Scenarios 1 and 2 with conventional and reduced tillage systems, respectively (Table 4). During the study years, wheat yields in Scenarios 3 and 4 were 9–36% higher than in Scenario 1 and 0–40% higher than in Scenario 2. Scenarios 1 and 2 did not differ in yield in the last three years of the experiment but the yield of Scenario 2 was 0.4 to 0.5 t ha^−1^ higher than that of Scenario 1 in the first two years (2009–10 and 2010–11).

**Table 4:** Grain yield of wheat during rabi season, rice or rice equivalent maize yield during kharif season, mungbean yield during summer season, and system-level yields (rice equivalent) under different scenarios during 2009–10 to 2013–14 in Karnal, India.

Scenario	2009–10	2010–11	2011–12	2012–13	2013–14	Overall^3^
	t ha^−1^					
Rabi/winter season (wheat)						
1	5.0b^1^	4.9c	6.0b	4.6b	4.6c	**5.0b**
2	5.5A	5.4b	5.9b	4.9b	4.5c	**5.2b**
3	5.5A	5.9a	6.5a	5.6a	6.3a	**6.0a**
4	5.5A	6.2a	6.8a	5.2ab	5.8b	**5.9a**
Average	*5.4*C**^4^	*5.6B*	*6.3A*	*5.1D*	*5.3CD*	

Kharif/rainy season (rice/maize)						
1	8.0A	6.1b	7.3b	6.8c	7.1b	**7.1b**
2	8.7A	7.2a	8.2a	7.9b	7.9a	**8.0a**
3	8.0A	7.4a	7.7b	5.6d	6.3c	**7.0b**
4	6.3B	7.2a	8.2a	9.4a	7.8ab	**7.7a**
	(7.1)^2^	(8.0)	(8.7)	(9.4)	(7.8)	**(8.2)**
*Average*	*7.74*AB	*7.00C*	*7.9A*	*7.4B*	*7.3B*	

Summer season (mungbean)						
1	–	–	–	–	–	–
2	0.7a	0.5a	0.3a	0.0b	0b	**0.3a**
3	0.0b	0.3b	0.1b	0.1a	0.1a	**0.1b**
4	0.0b	0.3b	0.1b	0.1a	0.1a	**0.1b**

System (rice equivalent)						
1	13.4b	11.2b	13.5c	11.5c	11.9c	**12.3c**
2	16.8a	14.5a	15.0ab	13.0b	12.6bc	**14.4a**
3	14.0b	14.6a	14.7b	11.5c	13.1ab	**13.6b**
4	12.2c	14.6a	15.6a	15.0a	13.9a	**14.3a**
Average	*14.1*B	*13.8C*	*14.7A*	*12.7D*	*12.9D*	

1 Within a column for each season and system, means followed by the same small letter are not different at the 0.05 level of probability.

2 Value in parentheses is original yield of maize crop.

3 Based on mixed model analysis for repeated measures by fitting covariance structure.

4 Within rows, means followed by the same capital letter are not different at the 0.05 level of probability.

**Fig. 2:**
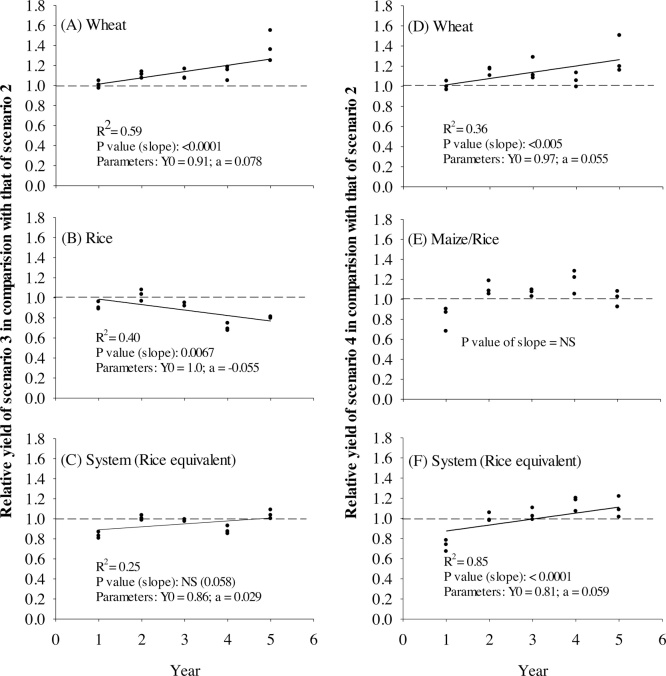
Relative yields of wheat, rice/maize, and system in Scenario 3 (A–C) and Scenario 4 (D–F) in comparison with those of Scenario 2 during the five study years. For trend analysis, linear regression is fitted. Values above the dotted line indicate higher yields than Scenario 2.

**Fig. 3:**
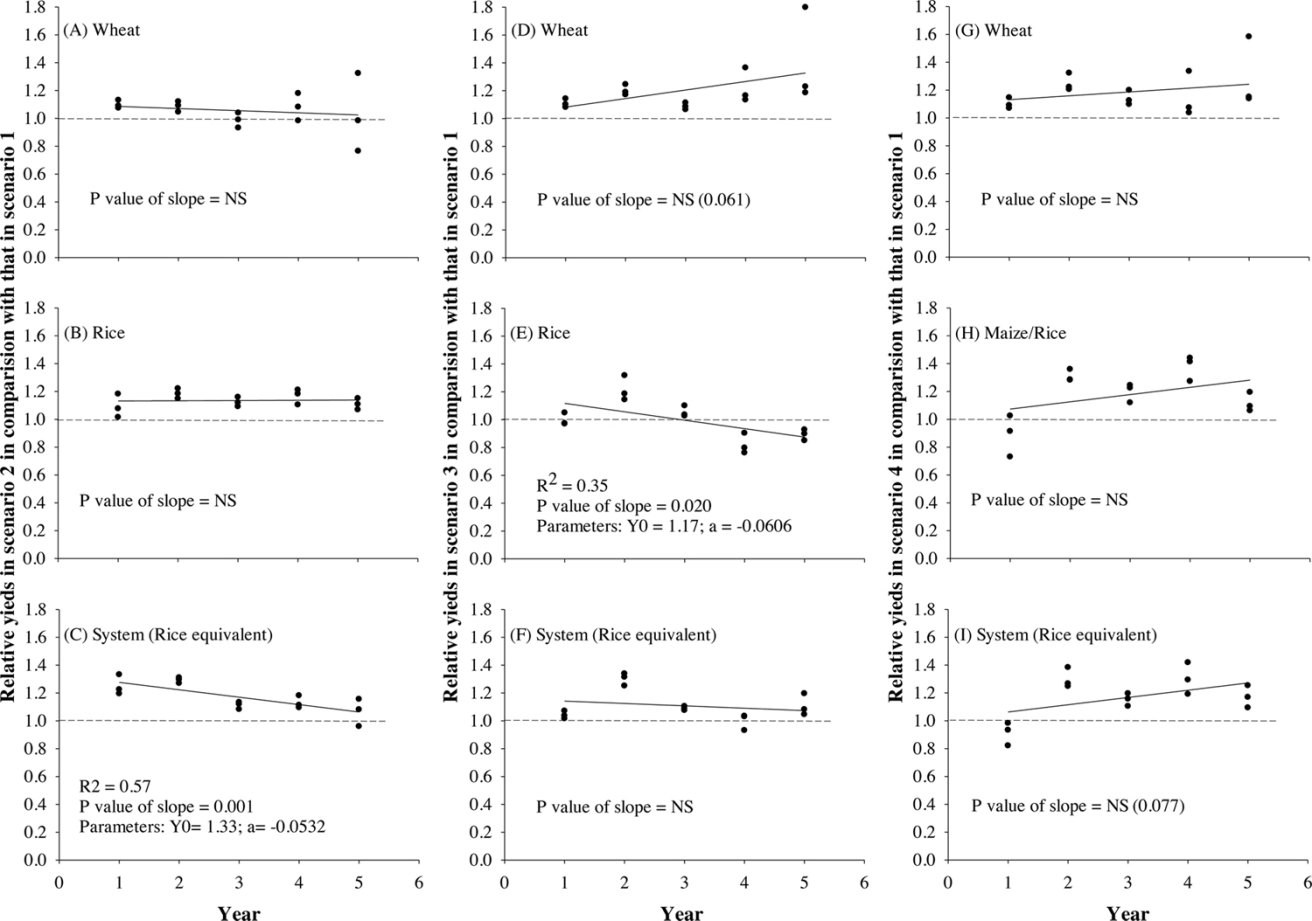
Relative yields of wheat, rice/maize, and system within Scenario 2 (A–C), Scenario 3 (D–F), and Scenario 4 (G–I) in comparison with those in Scenario 1 during the five study years. For trend analysis, linear regression is fitted. Values above the dotted line indicate higher yields than Scenario 1.

The relative yield of wheat in Scenarios 3 and 4 in comparison with that in Scenario 2 increased with time (Fig. 2A and D). Relative wheat yield in Scenarios 3 and 4 was approximately 1.0 in the first year but was >1.0 in the remaining four years, with a much higher value (1.3–1.4) in Year 5. In comparison to Scenario 1, the relative yields in Scenario 2 were more variable with no clear temporal trend (higher relative yield in Years 1 and 2 but similar in Years 3–5), whereas, in Scenario 3, relative yields showed a marginal increase with time (p value of slope = 0.061) (Fig. 3A and D). Relative yields were >1.0 (ranging from 1.1 to 1.4) in all the years in Scenarios 3 and 4, with the highest value in Year 5 (Fig. 3D and G).

Multiple management components differentiate the scenarios; hence, it is difficult to isolate which factors contribute to different wheat yield outcomes. The possible causes of higher yields in Scenarios 3 and 4 in comparison with the yield in Scenario 1 (Table 4; Fig. 3D and G) include a combination of early planting date (end of October versus mid-November), absence of puddling during the rice phase, ZT with residue retention, and inclusion of a legume in the system. Compared to Scenario 2, the possible causes of yield advantages and consistently higher relative yields in Scenarios 3 and 4 include a combination of early planting and the absence of puddling during the rice phase.

Early wheat sowing and ZT with residue retention have been reported to have a positive impact on wheat yield by mitigating the negative effect of terminal heat stress during wheat grain filling (Balwinder-Singh et al., 2016, CSISA, 2013, Gathala et al., 2011c). Simulations by Balwinder-Singh et al. (2016) predict that wheat yields of 15 November-sown wheat under mulched or non-mulched conditions are 0.8–1.0 t ha^−1^ and 0.4 t ha^−1^ lower than 31 October-sown wheat under mulched conditions in sandy loam and clay-loam soil, respectively, in Punjab, India. Early sowing minimizes the risk of terminal heat stress by completing the grain-filling phase before the onset of high temperatures, whereas ZT with residue mulch mitigates the effect of terminal heat stress by keeping the canopy cooler due to improved soil moisture retention (Gathala et al., 2011c). Gathala et al. (2011c) also observed a 9–10% higher yield under ZT combined with residue mulch compared with CT or ZT without residues. In addition, they noted that residue mulch reduced canopy temperature by 2–3 °C compared with CT during the late grain-filling stage. This finding suggests that residue mulch could be more beneficial in warmer years. In this study, higher yields were observed in Scenario 2 (ZT with residues) than in Scenario 1 (CT without residue) only in the first two years (Table 4 and Fig. 3A) when February and March were warmer than the long-term average and other study years during the grain-filling period. The monthly average maximum temperature (T_max_) in Year 1 (2009–10) was 2.2–2.9 °C and 3.3–5.3 °C higher in February and March, respectively, than in Years 3–5 (Fig. 1). The T_max_ of February and March 2009–10 was also higher than the long-term average by 0.7 and 3.3 °C, respectively. The monthly average minimum temperature (T_min_) of March in Year 1 was also 2.0–3.0 °C higher than the long-term average and in Years 3–5. T_max_ of Year 2 (2010–11) in February was 1.3–2.0 °C higher than in Years 3–5 but not from the long-term average, whereas T_min_ in February and March was slightly higher (0.6–0.7 °C) than the long-term average. These results suggest that CA (ZT with residue) increases resilience to the types of warm spring conditions that are anticipated to become more common with progressive climate change.

No yield advantages were observed in Scenarios 3 and 4 vis-à-vis Scenario 2 in the first year (Table 4 and Fig. 2A and D), which was expected since the experiment started with the wheat crop and sowing was done at the same time in all the scenarios in the first year. The reason for the increasing trend in relative wheat yields in Scenarios 3 and 4 over Scenario 2 (Fig. 2A and D) could be the combination of early planting and improvement in soil properties, especially better drainage associated with improved infiltration rate in continuous full CA practices (Jat et al., 2017). Improved drainage may have a given advantage for Scenarios 3 and 4 over Scenario 2 in Years 4 and 5, which were relatively wetter than the long-term normal and other study years (Fig. 1A). In addition, Scenarios 3 and 4 performed much better than Scenarios 1 and 2 in Year 5 as demonstrated by the much higher relative yield (1.3–1.4 t ha^−1^) than in the rest of the years (Fig. 2A and D). Wheat was planted considerably later in Scenarios 1 and 2 in Year 5 (November 30) than in other years (mid-November) (Table 1 in Supplementary data) and also higher rainfall conditions were observed in January to March than in the long-term average (Fig. 1A). Both factors likely contributed to the favorable yield outcomes relative to Scenario 2.

Averaging over all scenarios, the dominant influence of growing-season weather was apparent, with the highest average wheat yield (6.3 t ha^−1^) in Year 3 (2011–12) and the lowest in Year 4 (2012–13): 5.1 t ha^−1^. Variability across years corresponded to the average yields reported for Haryana State for the same period. High temperatures during grain filling in Year 1 (22.9 °C in February and 30.8 °C in March compared with 20–22 °C and 25.5–27.5 °C in February and March, respectively, in Years 2–5 and the long-term average) and less solar radiation and wetter seasons in Years 4 and 5 as compared to the long-term average and rest of the study years are likely causes of lower yields (Fig. 1). The most favorable combination of weather factors for high wheat productivity in Year 3 appears to be the combination of cooler temperatures during grain filling and high levels of solar radiation.

#### 3.2.2 Kharif crops: rice or maize

Grain productivity during the *kharif* season (expressed as REY) differed by scenario (Table 4). On the basis of the five-year average, Scenario 3 with ZT-DSR had a yield similar to that of PTR in Scenario 1. However, rice yields in Scenarios 1 and 3 were 0.92–0.98 t ha^−1^ lower than the yields in Scenario 2, where PTR was combined with BMPs. In REY terms, maize performance in Scenario 4 was similar to that of PTR with BMPs in Scenario 2 but was 10% higher (0.7 t ha^−1^) than in conventional PTR and ZT-DSR conditions in Scenarios 1 and 3.

The relative yield of rice in Scenario 2 in comparison with that in Scenario 1 ranged from 1.1 to 1.2 in all the years. In contrast, the relative yield of ZT-DSR in Scenario 3 in comparison with that of transplanted rice in Scenario 1 (Fig. 3E) and Scenario 2 (Fig. 2B) declined with time. The relative yield of maize in Scenario 4 compared with transplanted rice yield in Scenario 2 with BMPs was <1.0 in the first year but in the rest of the years was consistently either 1.0 or >1.0 (Fig. 2E), whereas, as compared with Scenario 1, except for year 1, the relative yields of maize were >1.0 in all years but varied greatly across the years (Fig. 3H).

The possible causes of higher yields of PTR in Scenario 2 compared with PTR in Scenario 1 include a combination of the use of younger seedlings, line transplanting, AWD water management, the inclusion of a legume crop in the system, and the incorporation of crop residues. Differences in yield because of the cultivar used are unlikely because the medium-duration hybrid (Arize 6444) and the long-duration high-yielding inbred (Pusa-44) used have similar yield potential. Moreover, in Year 5 (2013-14), the same cultivar (Arize-6129) was used in all rice scenarios and the yields were higher in Scenario 2 than in Scenario 1 (Table 4). Shah et al. (2011) observed a positive effect of incorporating mungbean and other legume green manure crops on rice yield in rice–wheat cropping systems compared with systems without legumes as legumes help improve soil organic carbon and soil fertility.

ZT-DSR plots maintained yields similar to those of PTR in the first three years; however, relative yield started to decline thereafter in comparison with that of transplanted rice in Scenarios 1 and 2 (Table 4; Fig. 2B and E). In Years 4 and 5, ZT-DSR yields were 10–18% and 20–30% lower than PTR yields in Scenarios 1 and 2, respectively. Some short-term studies conducted in India reported similar yields of DSR and PTR (Sudhir-Yadav et al., 2011a, Gathala et al., 2013, Kumar et al., 2015), while others reported lower yields in DSR than in PTR (Kumar and Ladha, 2011, Jat et al., 2009, Ladha et al., 2009, Saharawat et al., 2010). In the latter studies, the major explanations given for lower yields in DSR included (1) higher weed competition, (2) lack of suitable varieties, (3) suboptimal irrigation scheduling, and (4) higher spikelet sterility. In this present study, these are unlikely reasons for the lower yields obtained in Years 4 and 5 because weeds were effectively controlled, a suitable hybrid cultivar was selected, and tensiometer-based irrigation at a conservative threshold (15–20 kPa) was applied. Instead, the yield decline was probably because DSR experienced severe iron deficiencies in these years and, despite multiple foliar sprays of ferrous sulfate, the crops did not fully recover. Another reason behind the yield decline could be the presence of soil sickness and biotic stresses including nematode infestations as has been similarly reported by many other studies (Gathala et al., 2011a, Kreye et al., 2009). The more aerobic nature of DSR systems caused by AWD water management and the cumulative changes in soil physical properties that increase soil drainage are known causes of the reduced availability of micronutrients such as iron (Jat et al., 2017) while at the same time creating favorable conditions for nematode infestation. Analysis of soil samples collected at the end of the rice season after four years of study showed that available iron in Scenarios 3 and 4 was less than in Scenario 2 in upper (0 to 15 cm) and lower (16–30 cm) layers (Jat et al., 2017). The differences are likely to be larger early in the growing season when redox potential differs considerably between treatments.

These results raise important questions about the sustainability of ZT-DSR. In long-term experiments at the International Rice Research Institute in the Philippines, the yield of aerobic DSR declined over time compared with the yield in fully flooded fields due to nematode infestation and micronutrient deficiencies (Kreye et al., 2009, Peng et al., 2006). The long-term performance of continuous DSR also varied with soil type as a yield decline was observed earlier in lighter (sandy loam) soils (Bhatt and Kukal, 2015). In this study with loam soil, yield decline started after three seasons, whereas Jat et al. (2014) observed no yield decline in clay loam soil from Bihar, India, even after seven consecutive seasons of DSR. Some research findings have suggested that using cultivars with deep root systems, using the right source of N (ammonium sulfate) to mitigate increased soil pH associated with continuous aerobic DSR, and rotating aerobic DSR with fully flooded conditions may reverse the yield decline of continuous aerobic DSR systems (Nie et al., 2009a, Nie et al., 2009b, Nie et al., 2012).

In contrast to DSR, maize yield under CA increased over time in Scenario 4 with the exception of Year 5 (Table 4). Rice equivalent maize yields were lower in Year 1 (by 30% on average) but were either similar or higher than rice yields in subsequent years in Scenarios 1–3. Except in Year 1, maize yields were ≥8.0 t ha^−1^ with a maximum yield of 9.4 t ha^−1^ recorded in Year 4. The lower maize yield in Year 1 was probably because of higher rainfall than normal, which resulted in a delay in planting and more stress at the early stage of crop establishment due to waterlogging. In addition, solar radiation in August and September was relatively lower than the long-term average and in other study years. In subsequent years, ZT and residue retention resulted in improved soil physical properties such as infiltration rate (0.10 cm h^−1^ in Scenario 1 versus 0.30 cm h^−1^ in Scenario 4 after four years) that likely reduced waterlogging duration following rain events (Jat et al., 2017).

#### 3.2.3 Mungbean

Mungbean was included as an opportunity crop following the winter crop harvest. In Year 1, yield of 0.7 t ha^−1^ was obtained in Scenario 2 prior to PTR establishment but harvest was not possible in Scenarios 3 and 4 because the mungbean had to be killed before maturity to allow the timely planting of succeeding crops of DSR and maize, which are generally planted in the main field 2–4 weeks earlier than PTR. In the succeeding years, mungbean was relay-sown in the standing wheat crop 10–15 days prior to the wheat harvest to give a longer window to achieve crop maturity in Scenarios 3 and 4. With this management adjustment, grain yields were obtained in all the scenarios, with higher yield achieved in Scenario 2 (0.53 and 0.25 t ha^−1^ in Years 2 and 3, respectively) than in Scenarios 3 and 4 (0.3 and 0.1 t ha^−1^ in Years 2 and 3, respectively). In Years 4 and 5, when mungbean was drill-sown after the wheat harvest (the crop establishment method was changed from relay to drill sowing to achieve good and uniform establishment and higher biomass of mungbean, which otherwise was patchy in the relay seeding method), lower yields were achieved because of a combination of the limited time for mungbean to mature before the establishment of DSR and maize and the presence of more rain during flowering, which resulted in the faster vegetative growth of mungbean. In Scenario 2, in which sufficient time is typically available for one to two pickings, yield could not be obtained in Year 4 because of the appreciable amount of rainfall (157 mm) received during June. This much rainfall was sufficient to create a favorable condition for vegetative growth and suppressed the onset of the reproductive phase as the pulse variety (SML 668) showed indeterminate growth habits. Therefore, the pods were not harvested in Year 4 in Scenario 2. Similarly, in Year 5, regular rainfall in May, June, and July created a favorable environment for the vegetative growth of mungbean and the onset of the reproductive phase was postponed (15 days).

#### 3.2.4 System

On the basis of the five-year average, REY at the systems level varied in the following order: Scenario 2 = Scenario 4 > Scenario 3 > Scenario 1 (Table 4). Yields of the reduced tillage system with BMPs (Scenario 2) were higher by 0.7–3.4 t ha^−1^ in all five years than the yield in Scenario 1. Scenario 4 (full CA + strategic diversification) was 2.0–3.5 t ha^−1^ more productive than Scenario 1 except in Year 1. Also, the yields of Scenario 3 with full CA were either similar (in Years 1 and 4) or higher (in Years 2, 3, and 5) than that of Scenario 1. Compared to Scenario 1, rice yields in Scenario 3 declined in Years 4 and 5, but losses were offset by higher wheat yields, which resulted in similar system-level productivity. Compared to Scenario 2, the system productivity of full CA-based diversified cropping systems in Scenario 4 was similar but it was 6% lower in Scenario 3. Except in Year 1, the system yield of Scenario 4 was either similar to or higher than that of Scenario 2. In Year 1, the system yield in Scenario 4 compared with Scenario 1 was lower mainly because of lower maize yield and no mungbean yield. The reason for lower system yield in Scenario 3 than in Scenario 2 in the first years was mainly the difference in mungbean yield (no yield versus 0.7 t ha^−1^) (Table 4).

Time trend analysis shows that, as compared to Scenario 1, relative system yield in Scenario 2 was >1.0 but declined with time (Fig. 3C), whereas in Scenario 3 the relative yield varied greatly across years, ranging from 1.0 to 1.3 with no clear trend. Relative yield in Scenario 4 vis-à-vis Scenario 2 increased with time (Fig. 2F) and had a marginal increasing trend compared with Scenario 1 (Fig. 3; p value of slope = 0.077) with consistently relative yield of >1.0 in Years 2–4. The relative yields of Scenario 4 compared with Scenarios 1 and 2 were <1.0 in year 1, mainly because of lower maize yield in the first year (Figs. 2F, 3I; Table 4).

### 3.3 Water input and productivity

#### 3.3.1 Wheat

Based on the five-year average, irrigation water inputs in wheat did not differ between the full CA-based scenarios (3 and 4) and conventional management (Scenario 1) (Table 5). The amount of irrigation water was lowest in Scenario 2, which was 27 mm (7%) lower than in Scenario 3, but it did not differ statistically from Scenarios 1 and 4. In contrast, WP_I_ and WP_I+R_ of wheat differed significantly across scenarios (Table 6) and followed the same trend as wheat yield: Scenario 3 = Scenario 4 > Scenario 1 = Scenario 2. The WP_I_ and WP_I+R_ trends were variable across years; however, in most of the years, water productivity was higher in ZT wheat (Scenarios 3 and 4) than in CT wheat in Scenario 1 mainly due to the higher yields in Scenarios 3 and 4.

**Table 5:** Irrigation water application in each crop grown and at the systems level under four scenarios from 2009–10 to 2013–14 in Karnal, India.

Scenario	2009–10	2010–11	2011–12	2012–13	2013–14	Overall^2^	2009–10	2010–11	2011–12	2012–13	2013–14	Overall^2^
	Irrigation water (cm ha^−1^)						Total water input (irrigation + rainfall) (cm ha^−1^)					
Wheat												
1	42a^1^	43b	38c	28b	36a	37ab	44a^1^	51b	41c	48b	53a	47ab
2	44a	40c	41bc	30b	31b	37b	47a	48c	43bc	50b	48b	47b
3	41a	48a	47a	37a	27c	40a	44a	56a	49a	57a	44c	50a
4	40a	48a	45ab	36a	28bc	39ab	43a	56a	47ab	56a	45bc	49ab

Rice/maize												
1	194a	242a	237a	250a	216a	228a	270a	282a	280a	308a	252a	278a
2	135b	171b	163b	210a	192ab	174b	230b	219b	206b	268a	228ab	230b
3	134b	115c	121c	148b	163b	136c	234b	166c	164c	204b	206b	195c
4	13c	13d	39d	27c	33c	25d	88c	65d	82d	83c	76c	79d

Mungbean												
1	0b	0b	0b	0b	0b	0b	14b	23ab	0b	2b	14c	11b
2	24a	7a	16a	10a	9a	13a	29a	24a	16a	12a	23a	21a
3	24a	8a	17a	9a	10a	14a	29a	23bc	17a	12a	18b	20a
4	24a	7a	15a	9a	9a	13a	29a	22c	15a	12a	18b	19a

System												
1	236a	285a	275a	278a	252a	265a	328a	357a	321a	358a	318a	336a
2	204b	217b	220b	250a	232ab	225b	306b	291b	265b	330a	299a	298b
3	200b	170c	185c	194b	199b	190c	307b	244c	231c	272b	268a	264c
4	77c	69d	99d	72c	70c	77d	160c	143d	144d	150c	139b	147d

1 Within a column for each season and the system, means followed by the same letter are not different at the 0.05 level of probability.

2 Based on mixed model analysis for repeated measures by fitting covariance structure.

**Table 6:** Irrigation and total input water productivity of wheat, rice/maize, mungbean, and system grown under different scenarios during 2009–10 to 2013–14 in Karnal, India.

Scenario	2009–10	2010–11	2011–12	2012–13	2013–14	Overall^2^	2009–10	2010–11	2011–12	2012–13	2013–14	Overall^2^
	Irrigation water productivity (kg grain m^−3^)						Total input water productivity (irrigation + rain) (kg grain m^−3^)					
Wheat												
1	1.20b^1^	1.14c	1.57a	1.68a	1.27b	1.37b	1.13b^1^	0.96c	1.49a	0.96a	0.87b	1.08b
2	1.26Ab	1.35a	1.45a	1.64ab	1.48b	1.44b	1.19ab	1.13a	1.37a	0.99a	0.96b	1.13b
3	1.35A	1.24b	1.39a	1.52ab	2.36a	1.57a	1.27a	1.07b	1.33a	0.98a	1.44a	1.22a
4	1.36A	1.28ab	1.54a	1.47b	2.09a	1.55a	1.28a	1.10ab	1.46a	0.94a	1.30a	1.22a

Rice/maize												
1	0.41B	0.25b	0.31c	0.28b	0.34b	0.32b	0.30b	0.22c	0.26d	0.23b	0.29b	0.26c
2	0.64B	0.43b	0.51b	0.38b	0.42b	0.48b	0.38b	0.33c	0.40c	0.30b	0.35b	0.35b
3	0.60B	0.65b	0.64b	0.38b	0.39b	0.53b	0.34b	0.45b	0.47b	0.27b	0.31b	0.37b
4	4.96A	5.62a	2.12a	3.47a	2.36a	3.71a	0.71a	1.12a	1.01a	1.12a	1.01a	0.99a

Mungbean												
1	0.00B	0.00c	0.00c	0.00c	0.00b	0.00c	0.00b	0.00c	0.00c	0.00c	0.00b	0.00c
2	0.27A	0.76a	0.15a	0.00c	0.00b	0.24a	0.23a	0.22a	0.15a	0.00c	0.00b	0.12b
3	0.00B	0.39b	0.06b	0.06b	0.11a	0.12b	0.00b	0.14b	0.06b	0.05b	0.055a	0.06a
4	0.00B	0.42b	0.07b	0.07a	0.11a	0.13b	0.00b	0.14b	0.07b	0.06a	0.056a	0.06a

System												
1	0.57D	0.40d	0.49d	0.43c	0.48c	0.47d	0.41c	0.32d	0.42d	0.33c	0.39c	0.37d
2	0.82B	0.67c	0.74c	0.53bc	0.55c	0.66c	0.55b	0.50c	0.60c	0.40bc	0.42c	0.49c
3	0.70C	0.86b	0.88b	0.59b	0.66b	0.74b	0.46c	0.60b	0.69b	0.42b	0.49b	0.53b
4	1.58A	2.13a	1.86a	2.09a	2.02a	1.94a	0.77a	1.02a	1.20a	1.00a	1.01a	1.00a

1 Within a column for each season and the system, means followed by the same letter are not different at the 0.05 level of probability.

2 Based on mixed model analysis for repeated measures by fitting covariance structure.

The results of this study contrast with the findings of other published studies that reported irrigation water savings in ZT wheat compared with CT wheat (Bhushan et al., 2007, Gathala et al., 2011a, Gupta and Seth, 2007). The reason for no water savings or slightly higher water use in scenarios with full CA observed in this study could be the earlier planting (2 weeks) in Scenarios 3 and 4 when temperatures were higher. Moreover, it was expected in this study that irrigation water application would have been less in ZT wheat with residue retention as mulch if irrigation were applied based on SMP instead of at critical growth stages. A field study conducted by Balwinder-Singh et al. (2011a) found that, when irrigation in wheat was applied based on SMP criteria, residue mulch of 8 t ha^−1^ conserved soil moisture and hence delayed the application of irrigation, resulting in 75-mm savings of irrigation water, which is equivalent to one irrigation. Additionally, in their simulation work, they found that mulching would save one irrigation in 50% of the years (Balwinder-Singh et al., 2011b, Balwinder-Singh et al., 2016). These results suggest the need to optimize irrigation scheduling in wheat using SMP criteria in order to realize the potential irrigation water savings of CA-based systems.

#### 3.3.2 Kharif season: rice or maize

During the rainy season (rice/maize, five-year average), irrigation water input was highest in Scenario 1 in which PTR was managed with continuous flooding for much of the growing season, was intermediate in Scenarios 2 and 3 with AWD, and was lowest in Scenario 4, in which *kharif* maize with CA-based practices was grown instead of rice (Table 5). For rice, irrigation water input was 40% and 22% lower in Scenario 3 with ZT-DSR than in conventional PTR in Scenario 1 and PTR with AWD in Scenario 2, respectively. In all five years, irrigation water application was 24–52% (average 40%) lower in ZT-DSR (Scenario 3) than in PTR (Scenario 1). These results are also consistent with the findings of other studies that compared water application in DSR and PTR (Kumar and Ladha, 2011, Sudhir-Yadav et al., 2011b, Bhushan et al., 2007). For example, Kumar and Ladha (2011), in their meta-analysis of data from different countries, found water savings of an average of 21–25% with DSR compared with PTR. Similarly, Sudhir-Yadav et al. (2011b) in Punjab, India, observed 30–50% irrigation water savings in DSR compared with PTR when irrigation water was applied at 20 kPa SMP in both crop establishment methods without any yield penalty compared with continuous flooding. The lower irrigation water in ZT-DSR in Scenario 3 than in PTR with AWD water management in Scenario 2 could be explained by PTR with AWD being retained in a continuously flooded condition for the first 15–20 days after transplanting for good crop establishment as recommended for the transplanted system, whereas, in DSR, the fields were kept moist but not continuously flooded. In addition, ZT-DSR with residue mulch conserved soil moisture and delayed irrigation timing.

Within transplanted rice, 24% water savings were observed in Scenario 2 with AWD compared with Scenario 1. These results are consistent with other studies (Gathala et al., 2011a, Humphreys et al., 2010, Sudhir-Yadav et al., 2011b). Gathala et al. (2011a) in a field study in Modipuram, India, observed 22% irrigation water savings in transplanted rice with AWD. Humphreys et al. (2010) also reported irrigation water savings within the range of 15–40% in PTR with AWD water management.

Irrigation water application in maize (Scenario 4) was 86–89% lower than in rice in Scenarios 1–3. Overall, the WP_I_ of maize (based on REY) was 7–12 times higher than the WP_I_ of rice grown with different establishment methods in Scenarios 1–3 (Table 6). A similar trend was also observed for WP_I+R_ but the differences were much smaller. For example, the WP_I+R_ of maize was only 2.7 to 3.8 times higher than that of rice in Scenarios 1–3. The WP_I_ of maize over the years varied considerably and ranged from 2.12 to 5.62 kg grain m^−3^; however, WP_I+R_ over the years varied narrowly in the range of 0.7 to 1.1 kg m^−3^. The high water productivity in maize was mainly because of a drastic reduction in water application while maintaining yields similar to or higher than those of rice.

#### 3.3.3 Mungbean/fallow

During the summer (i.e., fallow or mungbean) season, irrigation water application was similar in Scenarios 2–4 where mungbean was cultivated (Table 5). Across years, irrigation water application was higher in Year 1 (244 mm ha^−1^) and Year 3 (160–170 mm ha^−1^) than in the rest of the years (70–95 mm ha^−1^) mainly because of much less rainfall in Years 1 and 3 during the cultivation period (43 mm in Year 1 and no rain in Year 3).

#### 3.3.4 System

Averaged over the five years of the experiment and calculated on an annual basis, irrigation water application and total water inputs (irrigation + rainfall) followed this sequence: Scenario 1 > Scenario 2 > Scenario 3 > Scenario 4 (Table 5). Compared to the conventional rice–wheat–fallow system in Scenario 1, irrigation water savings in the reduced till rice–wheat–mungbean systems in Scenario 2 and ZT for all three crops in Scenario 3 were 15% and 28%, respectively. The full CA-based rice–wheat–mungbean system (Scenario 3) had 16% irrigation water savings compared with the reduced-till rice–wheat–mungbean system (Scenario 2). In all the years, irrigation input was 15–40% lower in direct-seeded rice–wheat–mungbean systems with CA (Scenario 3) than in the conventional rice–wheat system in Scenario 1. With the substitution of rice with maize in Scenario 4, irrigation water application decreased by 71%, 66%, and 59% compared with Scenarios 1, 2, and 3, respectively.

At the systems level, overall (five-year average), WP_I_ decreased in the following order: Scenario 4 > Scenario 3 > Scenario 2 > Scenario 1. In all five years, WP_I_ of the system in Scenario 4 (2.8 to 5.4 times) was higher than in Scenarios 1 and 2. However, WP_I_ in Scenario 3 was higher in all years than in Scenario 1 and in most of the years except Year 1 than in Scenario 2. However, WP_I_ in Scenario 2 was higher than in Scenario 1 in three out of five years.

### 3.4 Economic analysis

#### 3.4.1 Wheat

Based on the five-year averages, the scenarios differed significantly in terms of production costs, net income, and B:C ratio (Table 7). The total production costs of ZT wheat in Scenarios 2–4 were lower by INR 5860–6353 ha^−1^ (13–15%) than in Scenario 1 with CT wheat. The net income of Scenarios 3 and 4 with early wheat sowing combined with full CA was higher than that of Scenario 1 (INR 15,856–16,094 ha^−1^; 34%) and Scenario 2 (∼INR 11,000 ha^−1^; 21%). The B:C ratio varied in the following order: Scenario 3 = Scenario 4 (2.65) > Scenario 2 (2.40) > Scenario 1 (2.07).

**Table 7:** Average production cost, net income, and benfit:cost ratio of wheat, rice/maize, and system under different scenarios in Karnal, India, based on 5-year average (2009–14).^1^

Scenario	Production cost (INR/ha × 1000)	Net income (INR/ha × 1000)	B:C ratio
Rabi/winter season (wheat)			
1	43.6a	46.7B	2.1c
2	37.3c	51.6B	2.4b
3	37.6bc	62.8A	2.7a
4	37.8b	62.6A	2.7a
Kharif/rainy season (rice/maize)			
1	45.8a	39.0d	1.9c
2	42.7b	53.3b	2.3b
3	37.9c	45.5c	2.2b
4	34.8d	67.5a	2.9a
System			
1	89.5a	85.8c	2.0d
2	88.2b	106.5b	2.2c
3	81.5c	106.5b	2.3b
4	78.3d	128.4a	2.6a

1 Within a column for each season and the system, means followed by the same small letter are not different at the 0.05 level of probability using Tukey’s HSD test.

#### 3.4.2 Kharif season: rice/maize

Overall, scenario effects were significant for total production costs, net income, and B:C ratio during the *kharif* season (Table 7). Production costs were lowest in Scenario 4 with ZT maize, followed by Scenario 3 with ZT-DSR and Scenario 2 with PTR combined with BMPs, and were highest in Scenario 1 with conventional PTR. Compared to Scenario 1, total production cost was INR 7950 ha^−1^ (17%) lower when rice was direct-seeded under ZT (Scenario 3) and was lower by INR 11,062 ha^−1^ (24%) when rice was diversified to *kharif* maize in Scenario 4. In contrast, net income was highest in Scenario 4 where maize was grown as an alternative to rice and was lowest in Scenario 1. Compared to Scenario 1, the net incomes of Scenarios 4, 3, and 2 were 72% (INR 28,475 ha^−1^), 16% (INR 6424 ha^−1^), and 36% (INR 14,228 ha^−1^) higher, respectively. B:C ratio declined in the following order: Scenario 4 > Scenario 2 = Scenario 3 > Scenario 1.

#### 3.4.3 System

At the systems level, Scenarios 2–4 had a lower cost of production than Scenario 1 (Table 7). The savings in cost of production in Scenarios 4, 3, and 2 compared with Scenario 1 were INR 11,116, 8000, and 1300 ha^−1^, respectively. Net incomes of all scenarios were higher than in Scenario 1. For example, Scenario 4 had 50% (INR 42,500 ha^−1^) higher net income, whereas Scenarios 2 and 3 had 24% (INR 20,700 ha^−1^) higher net income. The B:C ratio of the system followed the same trend as production costs. B:C ratio followed this trend: Scenario 4 > Scenario 3 > Scenario 2 > Scenario 1.

The higher net incomes and B:C ratios in Scenarios 2–4 in wheat, in rice/maize, or at the system level were due to the higher gross income associated with higher yield (Table 4) or to the lower production costs associated with CA-based practices (Table 7), or to a combination of both. Tillage and crop establishment (T&CE) practices constitute the major share of total production costs (Erenstein and Laxmi, 2008). In wheat, T&CE costs were INR 3420–4185 ha^−1^ lower in Scenarios 2–4 with ZT wheat than in Scenario 1 with CT wheat (Gathala et al., 2013). Similarly, the T&CE costs of ZT-DSR and ZT maize in Scenarios 3 and 4, respectively, were INR 6570–6930 ha^−1^ lower than for PTR in Scenario 1. At the system level when all crops were grown under ZT with full CA practices in Scenarios 3 and 4, T&CE costs decreased by INR 9900–10350 ha^−1^ compared to PTR, whereas this reduction was INR 4500 ha^−1^ in Scenario 2 in which partial CA was practiced. Many other researchers have also reported savings in T&CE costs and total input costs as well as higher net incomes in CA-based practices (Bhushan et al., 2007, Laik et al., 2014). Kumar and Ladha (2011), based on meta-analysis of data from India, estimated a reduction of USD 125 ha^−1^ in total input costs with ZT-DSR, which is similar to the findings of this study, in which savings of USD 122 ha^−1^ (INR 7950 ha^−1^) were found in ZT-DSR compared with PTR (Table 7). The rising cost of cultivation and declining profitability are some of the major reasons why farmers are seeking alternatives such as DSR or are diversifying to new crops such as maize, which requires fewer resources and less capital than rice.

### 3.5 Energy budgeting

#### 3.5.1 Wheat

In comparison with Scenario 1, the average annual energy input was 17% lower in Scenarios 2–4 (Table 8), mainly because of the 83–85% reduction in energy in T&CE in the ZT-based Scenarios 2–4 compared with CT wheat. Moreover, fertilizer input energy was also 6–7% lower in the full CA-based Scenarios 3 and 4 than in Scenario 1. Total energy outputs of wheat in Scenarios 3 and 4 were 10.7 to 19.2 GJ ha^−1^ higher than in Scenarios 1 and 2, mainly because of the higher grain and straw yields in Scenarios 3 and 4. These higher energy outputs and lower energy inputs in Scenarios 3 and 4 resulted in 15–19 GJ ha^−1^ higher net energy return than in Scenarios 1 and 2. These results are consistent with other published studies (Kumar et al., 2013, Laik et al., 2014). Kumar et al. (2013) in their study conducted in western Uttar Pradesh, India, found a 13% reduction in energy input (23,285 versus 20,244 MJ ha^‐1^) under ZT wheat compared with CT wheat. In the current study, savings in energy associated with fertilizer use with time in the CA-based Scenarios 3 and 4 were also observed, which are attributed to the retention of crop residues of rice, wheat, and mungbean and to the inclusion of a legume in the system, which resulted in lower N fertilizer application after 3 years (Table 1 in Supplementary data). Fuel consumption during land preparation and crop establishment also declined significantly (66–71%) in ZT-based systems (Scenarios 2–4) compared with CT-based Scenario 1. Electricity consumption followed almost the same trend as energy input for irrigation. It was highest in Scenarios 3 and 4 because of the slightly higher irrigation water application in these scenarios. Also, the specific energy of CT wheat was highest and that of full CA-based systems (Scenarios 3–4) was lowest.

**Table 8:** Estimated energy input (total, from tillage and crop establishment, irrigation, and fertilizer), energy output, net energy return, specific energy, fuel and electricity consumed in different scenarios during rabi (wheat), kharif (rice/maize), and at systems level in Karnal, India (based on 5-year average, 2009–14).^1^

Scenario	Total input energy	Total output energy	Energy input for T&CE	Energy input for irrigation water	Energy input for fertilizer	Net energy return	Specific energy	Fuel consumed	Electricity consumed
	GJ ha^−1^						MJ kg^−1^ grain	L ha^−1^	kWh ha^−1^
Wheat									
1	25.3a	165.6b	4.44A	5.4ab	11.4a	140b	5.1a	41a	450b
2	21.0b	160.0b	0.68B	5.4b	11.2b	139b	4.0b	12c	449b
3	20.9b	176.3a	0.80B	5.8a	10.6c	155a	3.5c	14b	485a
4	20.9b	179.2a	0.75B	5.7ab	10.7c	158a	3.6c	14b	478ab

Rice/maize									
1	53.9a	217.0c	3.90A	35.4a	12.5a	163c	7.7a	57a	2925a
2	40.7b	244.9b	2.72B	25.5b	10.4c	204b	5.1b	54b	2107b
3	35.3c	182.3d	0.61C	20.5c	12.0b	147c	5.2b	23c	1693c
4	18.1d	289.8a	0.43D	3.4d	12.3a	272a	2.2c	8d	278d

System (wheat + rice/maize + mungbean)									
1	79.2A	382.6d	8.34A	40.8a	23.9a	303d	6.6a	98a	3376a
2	65.5B	448.5b	3.78B	32.8b	21.6d	383b	5.0b	74b	2710b
3	59.6C	403.5c	1.79C	28.2c	22.6c	344c	4.6b	45c	2330c
4	42.3D	511.7a	1.55D	10.9d	23.0b	469a	3.1c	30d	906d

1 Within a column for each season and the system, means followed by the same small letter are not different at the 0.05 level of probability using Tukey’s HSD test.

#### 3.5.2 Rice/maize

In comparison with that of Scenario 1, the average annual energy input decreased by 24% in Scenario 2, by 34% in Scenario 3 with DSR, and by 66% when rice was diversified with maize in Scenario 4 (Table 8). This reduction occurred mainly because of the decrease in the energy required for tillage and crop establishment (30% in Scenario 2 and 84–89% in Scenarios 3 and 4), irrigation water (28%, 42%, and 90% in Scenarios 2, 3, and 4, respectively), and fertilizer (4% in Scenario 3 and 17% in Scenario 2). The scenarios varied in the following order for total energy output: Scenario 4 > Scenario 2 > Scenario 1 > Scenario 3. However, net energy return followed the following order: Scenario 4 > Scenario 2 > Scenario 1 = Scenario 3. The net energy return was 68–125 GJ ha^−1^ higher in Scenario 4 than in Scenarios 1–3, whereas net energy return was 41–57 GJ ha^−1^ higher in Scenario 1 than in Scenarios 1 and 3. Because of the high input energy in Scenario 1, specific energy was higher in this scenario than in the others and it was lowest when maize replaced rice in Scenario 4. Per hectare fuel consumption for tillage and crop establishment was 34 L lower in Scenario 3 and 49 L lower in Scenario 4 than in Scenario 1. Fuel consumption in Scenario 2 with best managed PTR was only 3 L lower than in Scenario 1. Electricity consumption followed the same pattern as irrigation: Scenario 1 > Scenario 2 > Scenario 3 > Scenario 4. Laik et al. (2014) also observed a 31% savings in energy requirement for DSR compared with PTR.

#### 3.5.3 System

In comparison with that of Scenario 1, the average annual total input energy was 17%, 25%, and 47% lower in Scenarios 2, 3, and 4, respectively (Table 8). This was attributed mainly to the savings in energy inputs for tillage and crop establishment, irrigation, and fertilizer in Scenarios 2–4 in wheat and rice/maize compared with Scenario 1. More specifically, savings in energy used in land preparation and crop establishment were 55%, 79%, and 81% in Scenarios 2, 3, and 4, respectively. Similarly, reductions in the energy used in irrigation water application were 20%, 31%, and 73% in Scenarios 2, 3, and 4, respectively. Fuel and electricity consumption followed the same trend. By diversifying rice with *kharif* maize in Scenario 4, savings in electricity ranged from 61% to 73% compared with those in scenarios with rice (Scenarios 1–3). At the systems level, overall savings in energy input in Scenarios 2–4 decreased slightly because of the additional energy required for mungbean production (Table 5).

### 3.6 Global warming potential (GWP)

#### 3.6.1 Wheat

The average seasonal GWP of ZT wheat in Scenarios 2–4 was 7–8% lower than that of CT wheat in Scenario 1 (Table 9). Diesel fuel (for land preparation and seeding), electricity (for irrigation water application), and fertilizers constitute the major share (78–82%) of the total GWP estimated for wheat, whereas emissions of GHGs (N_2_O and CH_4_) from the soil contributed only 14%. Diesel consumption was 27–29 L ha^−1^ lower in scenarios with ZT (Table 8). The GWP contribution of fertilizers was about 8% lower in Scenarios 3 and 4 compared with that in Scenario 1. This was mainly because, with time, fertilizer requirements decreased in CA-based scenarios (Table 1 in Supplementary data) in which crop residues were retained and mungbean was included in the system. In contrast, about 8% higher GWP from electricity use was observed in Scenario 3. Pesticides in wheat constitute a very small amount of the total GWP. There were no CH_4_ emissions in any of the treatments in wheat because the crop was cultivated in aerobic conditions during the dry winter season. For N_2_O emissions, the trend was similar to the GWP contributed by fertilizers. N_2_O emissions did not differ under CA and CT systems in this study at the same fertilizer rate; however, emissions increased with increases in N fertilizer (Padre et al., 2016). These results are consistent with the findings of Aryal et al. (2015), which reported a reduction in GHG emissions in ZT wheat compared with CT wheat. The GWP of wheat with CA practices might be further reduced if irrigation water could further be managed precisely using SMP criteria instead of being applied at fixed timings at critical growth stages.

**Table 9:** Estimated average GWP (total, due to diesel, electricity, fertilizer, pesticides, N_2_O, and methane emissions) of different scenarios during rabi (wheat) season, kharif (rice/maize) season, and at systems level based on 5-year average.^1^

Scenario	Total GWP	GWP by diesel	GWP by electricity	GWP by fertilizers	GWP by pesticides	N_2_O emissions	CH_4_ emissions
kg CO_2_ equivalent ha^−1^							
Wheat							
1	1608a	111	448	801	17	231	0
2	1501b	32	447	782	17	223	0
3	1482b	37	482	740	17	211	0
4	1481b	37	475	736	17	210	0

Rice/maize							
1	4713a	151	2908	862	62	409	320
2	3741b	143	2095	748	75	350	320
3	3209c	61	1683	854	71	401	140
4	2806d	20	276	882	51	1576	0

System (wheat + rice/maize + mungbean)							
1	6321a	262	3355	1663	79	640	320
2	5402b	193	2693	1529	92	573	320
3	4861c	116	2316	1590	88	611	140
4	4455d	75	900	1622	68	1788	0

1 Within a column for each season and the system, means followed by the same small letter are not different at the 0.05 level of probability using Tukey’s HSD test.

#### 3.6.2 Rice/maize

The experiment-wide GWP of rice was almost three times higher than that of wheat (Table 9). During *kharif*, the GWP of Scenarios 2, 3, and 4 was lower by 21%, 32%, and 40%, respectively, than in Scenario 1 (Table 9). CH_4_ emissions declined by 56% with the change in rice establishment from PTR in Scenario 1 or 2 to ZT-DSR in Scenario 3 as also reported by Padre et al. (2016). Moreover, CH_4_ emissions were eliminated with *kharif* maize. In contrast, N_2_O emissions were almost four times higher in maize in Scenario 4 than in rice. Electricity and fertilizer contributed 75–80% of the total GWP in rice, whereas GHG emissions from the soil contributed only 15–18%. In maize, the major contributors to the total GWP were the emissions of N_2_O from the soil (56%) followed by fertilizer input (31%). The GWP from diesel consumption was 60% lower when rice was directly sown with ZT in Scenario 3 than in conventional PTR (Scenario 1), which became 87% lower when ZT maize was grown instead of rice in Scenario 4. Similarly, the GWP from electricity was 28%, 42%, and 91% lower in Scenarios 2, 3, and 4, respectively. These results imply that more research is needed on N management in maize to reduce the GWP of this crop.

#### 3.6.3 System

On an annual basis at the systems level and in contrast to Scenario 1, the total GWP was lower by 15%, 23%, and 30% in Scenarios 2, 3, and 4, respectively. These differences are primarily attributable to differences during the *kharif* season (Table 9).

### 3.7 Multiple indicators, trade-offs, and prospects for sustainable intensification

**Fig. 4:**
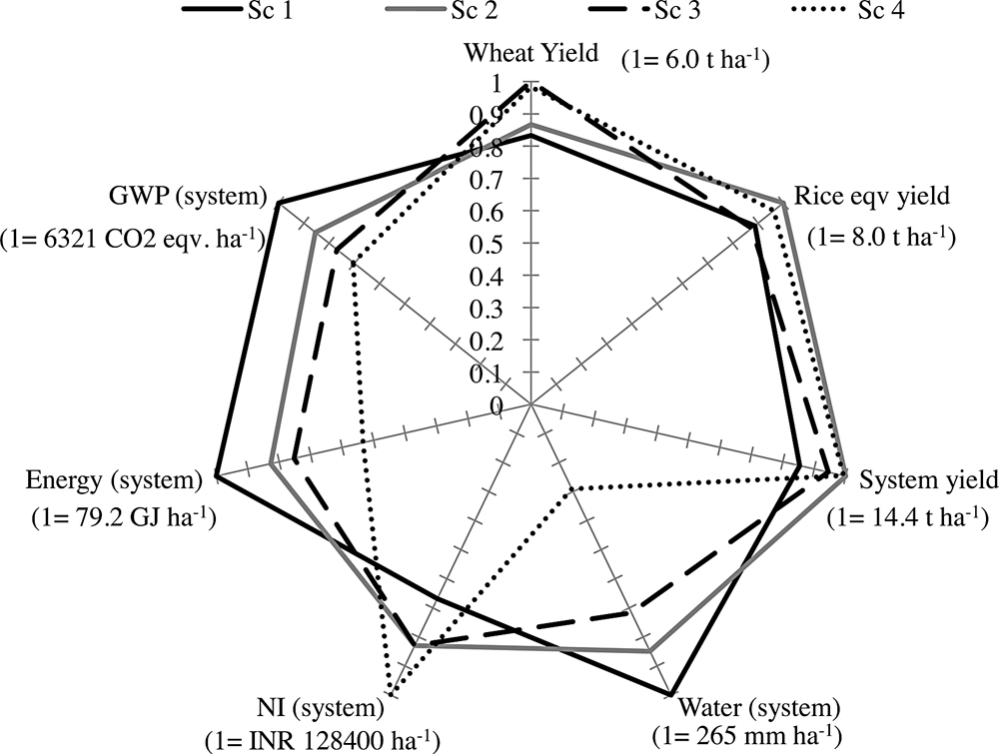
Multiple indicators of long-term performance of different scenarios. Performance metrics included wheat yield, rice equivalent yield in kharif season and system-level yield, irrigation water, net income, energy use, and global warming potential of cropping system scenarios in Karnal, India. Variable means are normalized on 0–1 scale, with 1 representing the highest absolute value of that variable. The highest absolute value is also shown for each parameter.

With the shift from the conventional rice–wheat–fallow system (Scenario 1) to reduced or CA-based rice or maize–wheat–mungbean system (Scenarios 2–4), a win–win situation was observed in all scenarios at the systems level without any trade-offs except at the crop level in Scenario 3 where rice yield declined in Years 4 and 5 (Fig. 4, Table 4).

As compared to Scenario 1, the reduced-till rice–wheat–mungbean system with BMPs (Scenario 2) resulted in higher system yield (17%) and profitability (24%) using 15% less irrigation water, 17% less energy input, and 15% less GWP (Fig. 4). As we shift from the reduced-till rice–wheat–mungbean system (Scenario 2) to a fully CA-based rice–wheat–mungbean system (Scenario 3), there was further savings of 15% in irrigation water, 9% in energy input, and 10% in GWP with similar profitability but with a trade-off of 0.8 t ha^−1^ yield penalty. However, the diversified CA-based maize–wheat–mungbean system (Scenario 4) provided higher net income (21%) than Scenarios 2 and 3 and similar (as compared to Scenario 2) or 5% higher (than Scenario 3) system-level yield using 59–66% less irrigation water input, 29–35% less energy input, and 8–18% less GWP than Scenarios 2 and 3. These results suggest that sustainable intensification (SI) is possible even in the most intensively cultivated areas of the IGP.

The ZT drill known as the Happy Seeder technology used in this study can directly seed wheat in the presence of full rice residue on the soil surface (Sidhu et al., 2007, Sidhu et al., 2015, Saunders et al., 2012). A major advantage of the technology is that it facilitates early wheat planting by reducing land preparation time because of directly drilling in ZT conditions while avoiding residue burning. This study clearly demonstrated the advantage of early wheat planting in wheat yield and ZT technology can facilitate harnessing this advantage by enabling early planting. In Punjab and Haryana states of India alone, about 17 million tons of rice residues are burned annually prior to wheat planting, resulting in the loss of nutrients, GHG emissions, and regional air pollution (Bijay-Singh et al., 2007, Singh et al., 2008, Saunders et al., 2012). Although a significant wheat area is already under ZT (e.g., 0.26 million ha in Haryana), most farmers practice it only after full or partial residue burning. More efforts are therefore needed to strengthen the service economy around the Turbo Happy Seeder equipment to increase the access of smallholder farmers to this capital-intensive technology.

During *kharif* season, this study demonstrated multiple options to achieve higher profitability and similar or higher yields with lower environmental footprint than the business-as-usual practice of flooded puddled transplanted rice (Scenario 1). For transplanted rice, irrigation water input, energy input, and GWP can be reduced to the extent of 20–24% by deploying AWD water management. The environmental footprint of rice can be further reduced by adopting direct-seeding of rice (>30% reduction in irrigation water input, energy input, and GWP) or by growing maize (90% reduction in irrigation water input and 40% reduction in GWP) as an alternative to rice under ZT (Tables 5, 8 and 9). If 10% of the total rice area in Haryana and Punjab (i.e., 0.4 million ha) is converted from rice to maize, the pumping of 60 million centimeters of groundwater for irrigation would be avoided, with an associated reduction of 5.1 to 9.6 million GJ of energy in pumping irrigation water and 0.76 Tg CO_2_ equivalent in GWP. Similarly, based on these estimates, switching rice establishment method from flooded PTR to ZT-DSR in Punjab and Haryana (4.0 million ha) might reduce GWP by 6.0 Tg CO_2_ eq. yr^−1^.

The inclusion of mungbean, a legume crop between two cereal crops, not only played a role in increasing system yield (Table 4, Fig. 4) but also has potential to carry out other ecosystem services, including improving soil heath (Jat et al., 2017) and minimizing weed problems in the following DSR crop (Rao et al., 2017). More research is needed to create a longer window for the inclusion of a legume and to identify mungbean cultivars that are better suited under relay cropping (cultivars that can establish better in the standing wheat crop), and are of determinate growth type that are less affected by rain during flowering to harness the full yield potential of mungbean. Because of these limitations, mungbean yields were low in our study and were quite variable (Table 4).

## 4 Conclusions

The key findings and conclusions of this study are summarized as follows:

1. This study clearly demonstrates that early wheat planting (by the end of October) can enhance wheat yields in NW India by mitigating the negative effects of terminal heat stress compared to when wheat is sown in mid-November, which is generally practiced by farmers and was considered optimum for the region in the past. For example, wheat yields were 0.7–0.8 t ha^−1^ higher when the crop was sown early (30 October) and combined with CA in Scenarios 3 and 4 than when sown around mid-November with ZT in Scenario 2.
2. ZT-DSR also appears to be an economically viable alternative to PTR to overcome the emerging problems of labor and water scarcity and rising production cost. When conventional PTR is replaced with ZT-DSR as in Scenario 3, irrigation water, energy use, and total GWP emissions all declined significantly by 40%, 34%, and 32%, respectively. Despite these advantages of DSR, however, some concerns about its long-term sustainability were raised by this study, especially if DSR is grown continuously, as evidenced by the yield decline in Years 4 and 5 compared to PTR. This calls for additional targeted research to understand the processes contributing to this decline.
3. Rice yields, profitability, and resource-use efficiency of conventional PTR can be enhanced by applying BMPs and incorporating mungbean in the crop rotation (Scenario 1 to Scenario 2).
4. *Kharif* maize under CA-based management appears to be a suitable and profitable alternative to rice, with even more significant reductions in irrigation water (89%), energy use (66%), and GWP emissions (40%) and with higher REY (0.7 t ha^−1^) and profitability (INR 28,475 ha^−1^) compared with conventional PTR. Despite these benefits, however, the adoption of maize as a diversification option for rice remains low in NW India. Current water and energy pricing policies as well as the public procurement system for rice (i.e., provision of a stable market) are disincentives for diversification.
5. Reduced-till or full CA-based rice–wheat–mungbean (Scenarios 2 and 3) and CA-based maize–wheat–mungbean systems have potential to increase the productivity (10–17%) and profitability (25–50%) of the rice–wheat–fallow system with less environmental footprint by using less irrigation water (15–71%) and energy input, and reducing total GWP (15–30%). These results provide strong evidence that the dual goals of enhancing productivity and profitability of cereal-based cropping systems in NW India can be reliably achieved while reducing environmental impacts.
