# Supplementary material for: Can productivity and profitability be enhanced in intensively managed cereal systems while reducing the environmental footprint of production? Assessing sustainable intensification options in the breadbasket of India
Source: Agric Ecosyst Environ. 2018 Jan 15;252:132–47. doi: 10.1016/j.agee.2017.10.006 (PMC5727681; doi:10.1016/j.agee.2017.10.006)
Supplement: Supplementary file 1 [file mmc1.docx]

**Appendix A. Supplementary data**

**Table 1.** Field operations and crop management details of rice, wheat, and maize under different scenarios during five study years (2009-10 to 2013-14).

| **Activity/operation** | **Scenario 1** | **Scenario 2** | **Scenario 3** | **Scenario 4** |
| --- | --- | --- | --- | --- |
| **Wheat** |  |  |  |  |
| Cultivar | PBW-343 in years 1, 2, 3; HD-2967 in years 4 and 5 | DBW-17 in years 1 and 2; HD-2967 in years 3, 4, and 5 | Same as Scenario 2 | Same as Scenario 2 |
| Field preparation | Two to three passes of each harrowing and cultivator followed by wooden planking | ZT | ZT | ZT |
| Sowing date | Yr. 1: 19 Nov.; Yrs. 2 & 3: 14-15 Nov.; Yr. 4: 22 Nov.; Yr. 5: 30 Nov. | Same as Scenario 1 | Yr. 1: 17 Nov.; remaining years: 30 or 31 Oct. | Same as Scenario 3 |
| Seed rate (kg ha^-1^) | 125 | 100 | 100 | 100 |
| Seed treatment | Similar in all scenarios (i.e., raxil) 2 DS (tebuconazole) @ 1 g kg^-1^ seed | | | |
| Sowing method | Manual broadcast, then seeds mixed in the soil using  Rotavator | Drill (line sowing) | Drill (line sowing) | Drill (line sowing) |
| Row spacing (cm) | Random | 20 | 20 | 20 |
| Nutrient management^1^ | Yrs. 1, 2, & 3: N-P_2_O_5_-K_2_O (150/172-58-0); Yrs. 4 & 5: N-P_2_O_5_-K_2_O (140/162-64-32) | Yrs. 1 & 2: N-P_2_O_5_-K_2_O (172-60-30); Yrs. 3 & 5 N-P_2_O_5_-K_2_O (150/162-64-32); Yr. 4: N-P_2_O_5_-K_2_O (111-64-32) | Yrs. 1 & 2: N-P_2_O_5_-K_2_O (172-60-30); Yr. 3: N-P_2_O_5_-K_2_O (150-64-32); Yrs. 4 & 5: N-P_2_O_5_-K_2_O (111/116-64-32) | Same as Scenario 3 |
| Weed management | Post-emergence herbicide (either sulfosulfuron + metsulfuron @ 32 g ai ha^-1^ or clodinafop-ethyl + metsulfuron @ 60 + 4 g ai ha^-1^ at 35 DAS | Same as Scenario 1 but no herbicide was applied in years 4 and 5 | Same as Scenario 1 but no herbicide was applied in years 4 and 5 | Same as Scenario 1 |
| **Rice/maize** | | | | |
| Cultivar | Pusa-44 (Yrs. 1, 2, 3, 4); Arize-6129 (Yr. 5) | Arize-6444 (Yrs. 1, 2, 3, 4); Arize-6129 (Yr. 5) | Arize-6129 in all years | NK-6240 (Yrs. 1, 2, 3); DKC-9125 (Yrs. 4 & 5) |
| Field preparation | Harrowing (x2), cultivator (x2) followed by wooden planking. Puddling done using harrow (x2), followed by planking. | Mungbean was killed by paraquat; then puddling was done using harrow (x3), followed by planking. | ZT (paraquat was used to kill mungbean and existing weeds) | ZT (paraquat was used to kill mungbean and existing weeds) |
| Crop establishment | Transplanting (random) | Transplanting (line) | Drill seeding | Drill seeding |
| *Date of sowing/transplanting* | 4-6 July (Yrs. 1, 2, 3, 4); 17 July (Yr. 5) | 25-28 June (Yrs. 1, 2, 3); 1 July (Yr. 4); 15 July (Yr. 5) | 18 June (Yr. 1); 7 June (Yr. 2); 10-12 June (Yrs. 3 and 4); 21 June (Yr. 5) | 6 July (Yr. 1); 7 June (Yr. 2); 12 June (Yr. 3); 21-23 June (Yrs. 4 and 5) |
| *Seed rate* | 15 | 12 | 22-25 | 22 |
| *Seed treatment* | Raxil 60 FS (tebuconazole) + Guicho 600 FS (imidacloprid) | Same as in Scenario 1 | Same as in Scenario 1 | Guicho 600 FS (imidacloprid) |
| *Method* | Transplanting (random) | Transplanting (line) | Drill (line seeding) | Drill (line seeding) |
| *Spacing* | Random | 20 cm x 15 cm | 20 cm | 60 cm x 20 cm |
| *Seedling age (days)* | 30-35 | 20-25 | Not applicable | Not applicable |
| Nutrient management^1^ | Yrs. 1, 2, & 3: N-P_2_O_5_-K_2_O (172-60-0); Yrs. 4 & 5: N-P_2_O_5_-K_2_O (168-64-32) | Yrs. 1, 2, & 3: N-P_2_O_5_-K_2_O (150-60-60); Yrs. 4 & 5: N-P_2_O_5_-K_2_O (138-64-32) | Yrs. 1, 2, & 3: N-P_2_O_5_-K_2_O (162-60-60); Yrs. 4 & 5: N-P_2_O_5_-K_2_O (138-64-32) | Yrs. 1, 2, & 3: N-P_2_O_5_-K_2_O (170/180-60-60); Yrs. 4 & 5: N-P_2_O_5_-K_2_O (170-64-32) |
| Weed management | Either butachlor @ 1000 g ai/ha or pretilachlor at 750 g ai/ha at 1 DAT, followed by (fb) 1 HW to remove escaped weeds | Same as in Scenario 1 | Pre-emergence (either oxadirgyl at 90 g ai ha^-1^ or pendimethalin at 1000 g ai ha^-1^) fb post-emergence herbicide depending on weed flora at 20-25 DAS. One HW was done to remove escaped weeds. | Atrazine either as PRE at 625 g ai ha^-1^ or early POST application at 10-15 DAS. In some years, tembotrione was applied as POST. One HW to remove escaped weeds. |

Table 2. Minimum support price and rates used for calculating costs of key inputs in economic analysis during different seasons.

| Particular | Year 1 | Year 2 | Year 3 | Year 4 | Year 5 |
| --- | --- | --- | --- | --- | --- |
| Minimum support price for wheat (INR kg^-1^) | 11.0 | 11.2 | 12.9 | 13.5 | 14.0 |
| Minimum support price for rice (INR kg^-1^) | 10.1 | 10.8 | 12.5 | 13.1 | 13.6 |
| Minimum support price for maize (INR kg^-1^) | 9.0 | 9.8 | 11.8 | 13.1 | 13.1 |
| Minimum support price for mungbean (INR kg^-1^) | 31.7 | 35.0 | 41.0 | 45.0 | 48.5 |
| Market price of wheat straw (INR kg^-1)^ | 3.0 | 3.0 | 3.0 | 5.0 | 5.0 |
| Diesel cost (INR L^-1^) |  |  |  |  |  |
| *Rabi season* | 36.5 | 36.5 | 40.0 | 45.7 | 51.4 |
| *Kharif season* | 36.5 | 40.0 | 40.0 | 49.3 | 56.2 |
| Labor wage (INR person^-1^ day^-1^) |  |  |  |  |  |
| *Rabi season* | 148.0 | 168.0 | 250.0 | 250.0 | 256.0 |
| *Kharif season* | 168.0 | 196.0 | 250.0 | 256.0 | 311.0 |
| Urea (INR kg^-1^) |  |  |  |  |  |
| *Rabi season* | 5.0 | 5.0 | 5.4 | 5.4 | 5.5 |
| *Kharif season* | 5.0 | 5.0 | 5.4 | 5.5 | 5.6 |
| DAP (INR kg^-1^) |  |  |  |  |  |
| *Rabi season* | 11.8 | 11.8 | 18.1 | NA | NA |
| *Kharif season* | 11.8 | 12.0 | 22.3 | NA | NA |
| NPK (12:32:16) (INR kg^-1^) | 11.2 | 11.2 | 16.4 | 21.0 | 21.0 |
| MOP (INR kg^-1^) | 5.0 | 5.0 | 5.0 | NA | NA |
| Zinc sulfate (INR kg^-1^) | 22.0 | 25.5 | 25.5 | 70.0 | 62.0 |
| Electricity charge (INR kWh^-1^) | 0.3 | 0.3 | 0.3 | 0.3 | 0.3 |
| Seed (INR kg^-1^) |  |  |  |  |  |
| *Wheat* | 35 | 35 | 35 | 35 | 35 |
| *Rice inbred* | 50 | 50 | 50 | 50 | 50 |
| *Rice hybrid* | 200 | 223 | 200 | 225 | 214 |
| *Maize hybrid* | 200 | 240 | 200 | 240 | 250 |
| NA: Not applied |  |  |  |  |  |
